# Synergistic mutations in soluble guanylyl cyclase (sGC) reveal a key role for interfacial regions in the sGC activation mechanism

**DOI:** 10.1074/jbc.RA119.011010

**Published:** 2019-10-23

**Authors:** Kenneth C. Childers, Xin-Qiu Yao, Sam Giannakoulias, Joshua Amason, Donald Hamelberg, Elsa D. Garcin

**Affiliations:** ‡ Department of Chemistry and Biochemistry, University of Maryland Baltimore County, Baltimore, Maryland 21250; § Department of Chemistry, Georgia State University, Atlanta, Georgia 30302-3965

**Keywords:** guanylate cyclase (guanylyl cyclase), nitric oxide, allosteric regulation, enzyme mutation, enzyme catalysis, molecular dynamics, cyclic nucleotide, enzyme mechanism, activation, luciferase assay

## Abstract

Soluble guanylyl cyclase (sGC) is the main receptor for nitric oxide (NO) and a central component of the NO-cGMP pathway, critical to cardiovascular function. NO binding to the N-terminal sensor domain in sGC enhances the cyclase activity of the C-terminal catalytic domain. Our understanding of the structural elements regulating this signaling cascade is limited, hindering structure-based drug design efforts that target sGC to improve the management of cardiovascular diseases. Conformational changes are thought to propagate the NO-binding signal throughout the entire sGC heterodimer, via its coiled-coil domain, to reorient the catalytic domain into an active conformation. To identify the structural elements involved in this signal transduction cascade, here we optimized a cGMP-based luciferase assay that reports on heterologous sGC activity in *Escherichia coli* and identified several mutations that activate sGC. These mutations resided in the dorsal flaps, dimer interface, and GTP-binding regions of the catalytic domain. Combinations of mutations from these different elements synergized, resulting in even greater activity and indicating a complex cross-talk among these regions. Molecular dynamics simulations further revealed conformational changes underlying the functional impact of these mutations. We propose that the interfacial residues play a central role in the sGC activation mechanism by coupling the coiled-coil domain to the active site via a series of hot spots. Our results provide new mechanistic insights not only into the molecular pathway for sGC activation but also for other members of the larger nucleotidyl cyclase family.

## Introduction

Soluble guanylyl cyclase (sGC)^5^ cyclizes guanosine 5′-triphosphate (GTP) into cyclic guanosine 3′,5′-monophosphate (cGMP), which controls vasodilation and platelet activity (1, 2). Nitric oxide (NO) binding to the N-terminal heme cofactor dramatically increases production of cGMP, which acts as a second messenger to regulate cardiovascular function. Impaired NO signaling and cGMP production have been linked to a variety of diseases (3). The predominant isoform of sGC is the α_1_β_1_ heterodimer (GC-1) with each subunit composed of four domains: an N-terminal regulatory heme-nitric oxide/oxygen binding (HNOX) domain, followed by a Per-Arnt-Sim (PAS) domain, an extended coiled-coil (CC) domain, and a C-terminal guanylyl cyclase (GC^cat^) domain (4). How the NO-binding event is transmitted from the regulatory domain to the catalytic domain remains unknown.

Although there are several structures of domains homologous to those present in sGC (5–11), only two structures of human αβGC^cat^ in inactive conformations have been solved so far (12, 13). Despite extensive efforts to obtain an active αβGC^cat^ structure, our understanding of the mechanisms by which αβGC^cat^ transitions to an active conformation relies principally on comparisons with the homologous adenylyl cyclase (AC) catalytic domains for which a wealth of structural and mutagenesis data are available (14–22). The activating events triggered by NO binding likely include a rearrangement of the two subunits to close the GTP-binding cleft (12). Several studies have suggested a possible assembly for the full-length enzyme and generated new hypotheses regarding the mechanisms by which the NO signal is transmitted to the active site (23–26).

For GC-1, only two activating mutations have been reported thus far, and both are located in the catalytic domain. The αC595S mutation located at the dimer interface (27) and the βM537N mutation located on the dorsal flap (28) both increased basal activity 7-fold compared with WT sGC. Although the mechanisms by which these mutations activate sGC remain unknown, we previously proposed that they modulate interfacial contacts to promote an optimal conformation of αβGC^cat^ for catalysis (13).

Identification of novel activating mutations will allow us to determine amino acids and structural elements important for sGC activation. Here, we describe the design, optimization, and utilization of a cGMP-based luciferase reporter assay to identify activating sGC mutations. This is the first time that such an assay has been designed to report on heterologous sGC activity in *Escherichia coli*. By combining measurements of luciferase activity and extracellular cGMP/cAMP levels, we identified several activating mutations in the catalytic domain. Combination of some of these mutations leads to synergism or antagonism, suggesting a complex cross-talk between various structural elements in the catalytic domain. Interestingly, our assay allowed us to identify mutants that affect not only cGMP synthesis, but also the ATP cyclase activity of sGC and its substrate specificity. We used molecular dynamics (MD) to further validate our experimental results. These studies support the key role of the dorsal flaps in activation and highlight additional interfacial regions impacted by activating mutations. Based on our results, we propose that the NO signaling event is communicated to the catalytic domain via hot spot linkages that couple the final helix-turn-helix motif of the coiled-coil domain to the active site. To our knowledge, this is the first time that synergy among various regions is discovered for enzymes that belong to the larger class of nucleotidyl cyclases. Importantly, targeting these regions with small molecules may provide new strategies to rationally design novel sGC activators.

## Results

### Assay promiscuity and background reduction

To identify novel activating mutations, we designed, optimized, and validated a luciferase reporter assay that measures heterologous sGC activity in bacterial cells. For this assay, we co-transformed *E. coli* cells with pCDF-αβGC1 (expressing heterodimeric GC-1 constructs), pGro7 (expressing GroEL/ES chaperones), and pOPTXcGMPRE:LUC (expressing Firefly luciferase) (29) plasmids (Table S1). Induction of sGC expression in the presence of GroEL/ES chaperones will produce cGMP, which in turn will induce expression of luciferase. Luciferase activity measurements will therefore be an indirect measure of cGMP levels and sGC expression and activity in *E. coli*. We optimized multiple cell growth parameters including isopropyl β-d-1-thiogalactopyranoside concentration, temperature, and growth time, as well-as volumes of harvested samples and lysis buffer to perform the luciferase assay.

In BL21(DE3) cells transformed only with the pOPTXcGMPRE:LUC vector, we measured significant luciferase activity (Fig. S1). Because *E. coli* cells do not produce significant amounts of cGMP (30, 31), we hypothesized that this was most likely due to a promiscuous promoter, which could also respond to endogenous cAMP production (29), or to other nucleotides. To reduce background luciferase activity, we tested BL21(DE3) cells lacking the *cyaA* gene, which encodes for endogenous adenylyl cyclase (32–34). Comparison of background luciferase activity in *cyaA*^+^ or *cyaA*^−^ cells in the absence of pCDF-αβGC1 and pGro7 revealed a ∼90% decrease when endogenous AC was absent (Fig. S1). As previous work indicated *E. coli* secretes a majority of cyclic nucleotides into the cell culture medium (33), we confirmed that decrease in luciferase activity in *cyaA*^−^ cells was due to the loss of cAMP production by measuring extracellular cAMP levels via immunoassays in both cell lines (Table S2). In addition, we confirmed that cGMP was undetectable in BL21(DE3) *cyaA*^−^ cells in the absence of the GC-1 expression vector. The residual luciferase activity in BL21(DE3) *cyaA*^−^ cells transformed without the GC-1 expression vector (pOPTXcGMPRE:LUC alone) was then likely due to cyclic nucleotides present in the cell culture medium. It is also possible that the plant OPTXcGMPRE promoter responds to metabolites other than cGMP/cAMP that could be present inside the cell or in the culture medium, as the exact mechanism of action of this plant promoter in bacterial cells is not known (29, 35). Our attempts to use minimal media to further eliminate background luciferase activity were unsuccessful. In light of this, we determined a background threshold for luciferase activity in the presence of the pGro7 and pCDF-αGC1 (lacking βGC-1) plasmids. This background was subtracted from all subsequent luciferase activity measurements to reveal only heterodimeric GC-1 construct-specific activity. Mutants with impaired activity were not pursued. We performed Western blotting analyses to confirm that increased activity was not due to differences in protein expression levels (Fig. S2).

### Luciferase activity induction by WT GC-1 and inhibition by inactivating GC-1 variants

To test the feasibility of the novel reporter assay, we measured luciferase activity in BL21(DE3) *cyaA*^−^ cells co-transformed with WT pCDF-αβGC1, pGro7, and pOPTXcGMPRE:LUC vectors. Samples were collected at several time points and luciferase activity was measured in clarified cell lysates. Detectable activity was defined as measurements with RLU/mg of protein levels greater than background (Fig. S3). Luciferase activity was only detectable in these cells 72 h post-induction (Table 1, Fig. S4). The reason for this is unknown, but it could be due to the slow growth conditions (15 °C, 90 rpm). To confirm these findings, we determined intracellular (cell lysates) and extracellular (supernatant) cGMP levels 72 h post-induction, which showed elevated cGMP levels mostly in the supernatant (Table 1, Table S3). The cGMP measurements support the luciferase assay results and demonstrate the utility of the reporter assay. For all subsequent experiments, only cGMP/cAMP levels measured for samples at 72 h post-induction are reported in Table 1. Luciferase activity was measured at various time points (see supporting data), but only the 72-h time point measurements are reported in Table 1 for consistency.

**Table 1:** **Luciferase activity and extracellular cGMP/cAMP levels for wildtype and mutant GC-1** Luciferase assay was measured in cell lysates from cell pellets collected after 72-h cell growth and were plated in triplicate. The cGMP and cAMP levels were measured in the extracellular supernatant after 72 h of cell growth and were plated in duplicate. Error represents the mean ± S.E. from three or more independent experiments. For luciferase activity, statistical significance (*p* value) between wildtype and mutant GC-1 samples was calculated using the Student's *t* test.

Sample	Luciferase activity	Fold-increase	*p* value	pmol cGMP/mg protein (× 10^3^)	Fold-increase	pmol cAMP/mg protein (× 10^3^)	Fold-increase	cGMP/cAMP ratio
	RLU/mg protein							
αGC-1 (-βGC-1)				ND*^a^*		N/D*^a^*		
Wildtype αβGC-1	274 ± 86	1.0		1.9 ± 0.5	1.0	0.6 ± 0.1	1.0	3
**α-Flap variants**								
αV587I/αV589T	1220 ± 229	4.5	*^b^	3.8 ± 0.9	2.0			
αV587I/αV589T/αK590R	667 ± 165	2.4		3.7 ± 1.1	2.0			
αM591N	1264 ± 206	4.6	*	3.7 ± 0.5	2.0			
**β-Flap variants**								
βI533M	1298 ± 245	4.7	*	2.9 ± 1.4	1.5			
βM537N	738 ± 132	2.7		4.2 ± 0.9	2.2			
βP538Q	1244 ± 331	4.5		2.7 ± 0.8	1.4			
**αβ-Flap variants**								
αV587I/V589T/K590R/βM537N	1062 ± 72	3.9	***	3.5 ± 2.0	1.9			
αβ Flap deletions	1688 ± 101	6.2	***	3.2 ± 0.8	1.7			
αM591N/βM537N	2623 ± 496	9.6	*	4.5 ± 1.2	2.4			
**Interfacial variants**								
αC595S	579 ± 135	2.1		12.2 ± 2.0	6.5			
αC595S/βT474V	2668 ± 463	9.7	**	6.4 ± 1.3	3.4			
βT474V	1250 ± 311	4.6		3.9 ± 1.4	2.1			
βT474M	1726 ± 381	6.3	**	4.4 ± 0.5	2.4			
αC595S/αE526A	3366 ± 1315	12.3		9.4 ± 1.5	5.0			
αC595Y	677 ± 5	2.5	*	0.5 ± 0.3	0.3	1.3 ± 0.4	2.2	0.4
**Interfacial/β-flap variants**								
αC595S/βM537N	965 ± 246	3.5		19.5 ± 4.1	10.4			
αC595S/βM537N/βP538Q	1925 ± 241	7.0	**	9.2 ± 2.3	4.9			
αC595S/βP538Q	439 ± 124	1.6		2.0 ± 0.8	1.1			
**GTP cleft variants**								
βN548W	ND	—		ND	—			
βC541G	1802 ± 374	6.6	*	4.1 ± 1.2	2.2	4.5 ± 1.3	7.6	0.9
**Interfacial/GTP cleft variants**								
βC541G/αC595S	2060 ± 190	7.5	***	24.8 ± 1.4	13.2	3.7 ± 1.5	6.2	6.7
**Interfacial/β-flap/GTP cleft variants**								
αC595S/βM537N/βN548W	ND	—		1.5 ± 0.3	0.8			
βC541G/αC595S/βM537N	1958 ± 344	7.1	**	28.6 ± 4.6	15.3	3.1 ± 0.8	5.3	9.0
βC541G/αC595S/βM537N/βP538Q	297 ± 70	1.1		10.0 ± 1.7	5.3	1.8 ± 0.4	3.0	5.6

*^a^* ND, not detectable.

*^b^* Statistical significance (*p* value) between wildtype and mutant GC-1 samples was calculated using the Student's *t* test: *, *p* < 0.05; **, *p* < 0.01; ***, *p* < 0.001.

**Figure 1.**
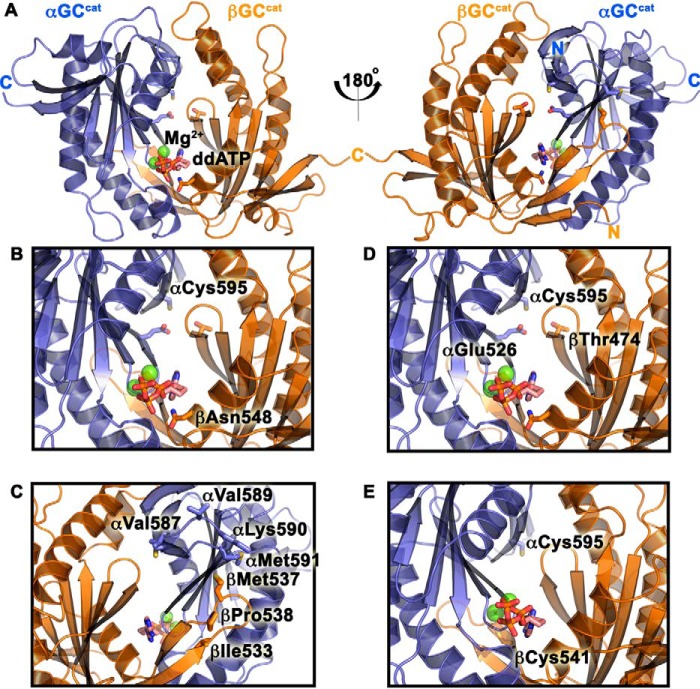
**Residues in the GC-1 catalytic domains targeted for mutagenesis.** *A,* ventral (*left*) and dorsal (*right*) sides of the GC-1 catalytic domains (αβGC^cat^) in the modeled activated conformation with bound Mg^2+^ and dideoxy-ATP (*ddATP*) (13). *B,* residues αCys-595 and βAsn-548 were mutated to inactivate GC-1. *C,* dorsal flaps residues were mutated. *D,* residues αCys-595, αGlu-526, and βThr-474 were predicted to form a hydrogen-bond triad (13). *E,* substrate-binding residue βCys-541 was proposed to modulate substrate specificity (38).

To determine whether we could specifically reduce luciferase activity by inactivating sGC, we made the αC595Y variant (Fig. 1, *A* and *B*), which was previously reported to reduce catalytic activity (28). Surprisingly, cells transformed with this variant showed luciferase activity 2.5-fold higher than cells transformed with WT GC-1 (Table 1, Fig. S4). However, we showed that these cells displayed 70% reduced extracellular cGMP levels, in good agreement with previous studies. We hypothesized that the higher luciferase activity may be caused by weak AC activity of mutant GC-1. Indeed, extracellular cAMP measurements for cells transformed with the αC595Y GC-1 variant were 2.2-fold higher than those transformed with WT GC-1, confirming our hypothesis. How this substitution modifies GC-1 substrate specificity is not clear, but it is possible that the bulky Tyr residue at the dimer interface modifies the nearby GTP-binding pocket to favor ATP binding instead.

To further inactivate GC-1, we made the novel βN548W variant (Fig. 1*B*). Based on the αβGC^cat^ crystal structure (PDB code 4NI2) and comparison with AC (12, 13), we hypothesized that mutation of βAsn-548 into a bulky Trp would hinder nucleotide binding and inhibit catalytic activity. Indeed, cells expressing βN548W GC-1 had no detectable luciferase activity or extracellular cGMP levels (Table 1), despite expression levels similar to WT GC-1 (Fig. S2).

Together, these results confirm that the luciferase assay specifically reports on heterologous GC-1 activity, and that its cGMP/cAMP production can be deconvoluted with follow-up ELISA measurements.

### Variants along catalytic domain dorsal flaps modulate GC-1 activity

To determine the dynamic range of the assay, we attempted to activate GC-1 with various known strategies. The most relevant of these is using the natural activator, NO, to activate full-length sGC. Due to the necessity of an N-terminal expression tag on the βGC-1 subunit in our construct (MoCR), we expected this to preclude NO-sensitivity of sGC (36). Indeed, when we exposed our system to various NO-donating molecules (NOR-3, NOC-18, and DEA-NONOate), no increase in luciferase activity was observed, suggesting that GC-1 was not activated. Consequently, we transformed cells with a known activating GC-1 variant for which we expected to see an increase in luciferase activity. The βM537N mutation, which is located on the dorsal flap of the βGC^cat^ subunit (Fig. 1*C*), was shown to activate GC-1 (28). Indeed, cells expressing the βM537N variant showed increased luciferase activity at multiple time points (Table 1, Fig. S5), in contrast to cells expressing WT GC-1. Cells expressing the activating variant had 2.7-fold greater luciferase activity and 2.2-fold greater extracellular cGMP levels than those expressing WT GC-1 (Table 1). Although the increase in luciferase activity may seem modest, it is significant (*p* value is 0.054). Additionally, the results of the luciferase activity measurements matched those obtained for cGMP levels. These results validate the reporter assay in detecting activating mutations in GC-1. We then set out to identify other potential activating variants in both GC-1 dorsal flaps.

First, we made the βI533M and βP538Q variants (Fig. 1*C*) along the βGC^cat^ dorsal flap, which correspond to activating I1010M and P1015Q mutations in AC (37). We observed activity profiles similar to cells expressing the βM537N variant with measurable luciferase activity at multiple time points (Table 1, Fig. S5) and moderately increased cGMP levels compared with cells expressing WT GC-1 (1.5- and 1.4-fold increase, respectively).

Second, we made a series of mutations in the αGC^cat^ dorsal flap. Cells expressing the αM591N variant, which is homologous to βM537N (Fig. 1*C*), also displayed luciferase activity at multiple time points. These cells showed 4.6-fold greater luciferase activity and 2.0-fold higher cGMP levels than those expressing WT GC-1 (Table 1). In cells expressing the double αM591N/βM537N variant, we measured luciferase activity and extracellular cGMP levels that were 9.6-fold and only 2.4-fold higher, respectively, than cells transformed with WT GC-1 (Table 1, Fig. S5). There was no apparent additive effect of the double mutation and the discrepancy between the luciferase activity and cGMP levels was likely due to increased AC activity, although it was not measured for the double variant. Thus, our luciferase reporter assay and cGMP measurements revealed the novel activating dorsal-flap αM591N mutation.

Third, to mimic the βGC^cat^ dorsal flap, we made double αV587I/V589T and triple αV587I/V589T/K590R variants (Fig. 1*C*). We hypothesized that these mutations may increase the number of contacts between the αGC^cat^ flap and the βGC^cat^ subunit to promote the “double flap-wrap” conformation observed for most AC structures (13). Cells expressing αV587I/V589T and αV587I/V589T/K590R variants displayed measurable luciferase activity at multiple time points, and 4.5- and 2.4-fold greater luciferase activity than cells expressing WT GC-1, respectively. Their extracellular cGMP levels were 2.0-fold greater than those expressing WT GC-1 (Table 1). This activity was similar to what we observed with cells expressing other variants along the GC-1 dorsal flaps, confirming a regulatory role for the dorsal flaps.

**Figure 2.**
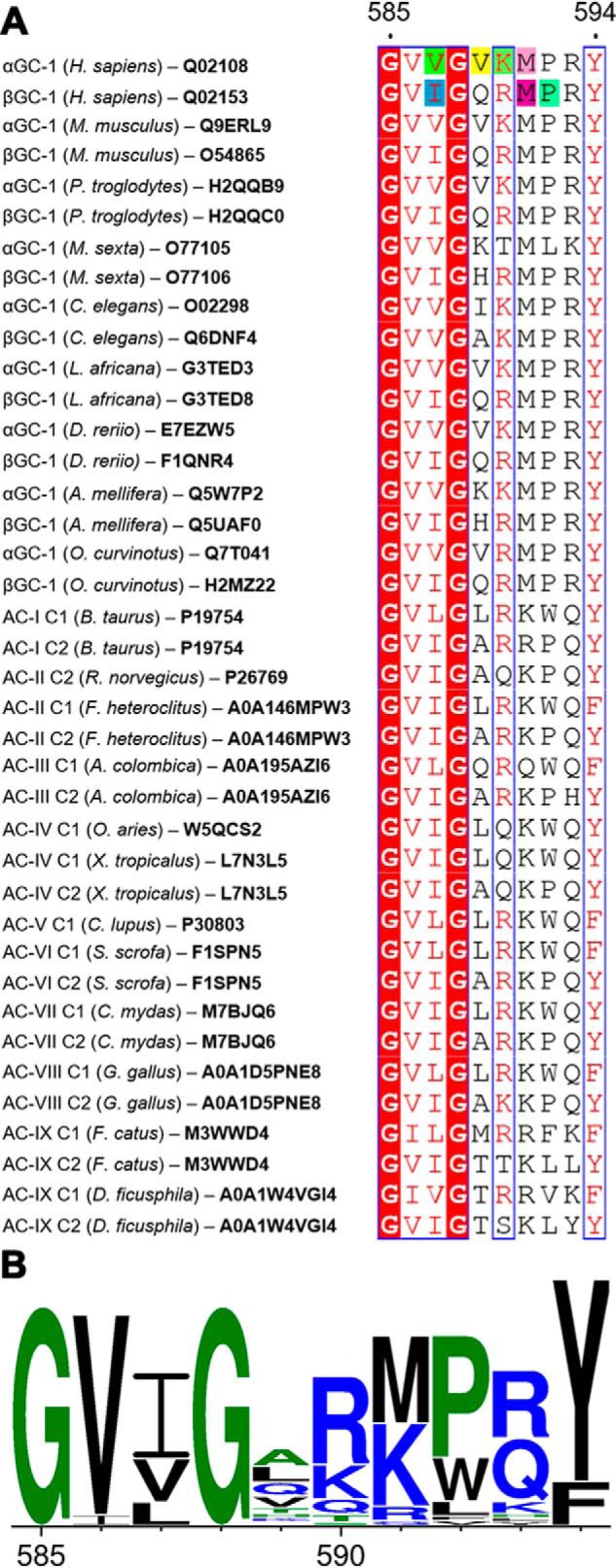
**Alignment of guanylyl cyclase and adenylyl cyclase dorsal flaps.** *A,* alignment generated with CLUSTAL Omega (71–73) and visualized with ESPript (74, 75). Numbering corresponds to the dorsal flap sequence for αGC-1 (*Homo sapiens*). Similar residues (*red letters*) are in *blue boxes* and invariant residues are highlighted in *red*. Residues in *H. sapiens* GC-1 that were mutated in this study are highlighted as follows: αVal-587 (*green*), αVal-587 (*yellow*), αLys-590 (*pale green*), αMet591 (*pink*), βIle-533 (*blue*), βMet-537 (*purple*), and βPro-538 (*teal*). Uniprot accession codes are indicated for each sequence. *B*, the graphic representation of conserved residues was generated with WebLogo (76).

Finally, our alignment of the dorsal flaps in GC and AC enzymes revealed two AC enzymes without dorsal flaps (Fig. 2). As a result, we sought to determine the effect of removing both dorsal flaps on GC-1 activity. We replaced αGC^cat^(588–592) and βGC^cat^(534–538) residues with Ala-Gly dipeptides. Surprisingly, cells expressing these “flap-less” variants showed activities very similar to those expressing the dorsal flap variants described above. Luciferase activity and extracellular cGMP levels were 6.2- and 1.7-fold greater, respectively, than cells expressing WT GC-1 (Table 1). Overall, our results suggest that all the mutations described above may alter the balance of contacts between the flaps and other GC-1 domains, and play a key role in the activation mechanism.

### Variants at the dimer interface activate GC-1 and synergize with dorsal flaps variants

Previous reports showed that the αC595S mutant displayed a 7-fold increase in basal GC-1 activity (27). Accordingly, we showed that cells expressing αC595S GC-1 yielded a 2.1-fold increase in luciferase activity and a 6.5-fold increase in cGMP levels compared with cells expressing WT GC-1, which matches closely previous levels in a different cell system (28). Surprisingly, cells expressing the double αC595S/βM537N variant showed increased luciferase activity (3.5-fold) and dramatically increased cGMP levels (10.4-fold) over cells expressing WT GC-1. This synergistic activation was almost completely abolished in cells expressing the triple αC595S/βM537N/βN548W variant, showing the deleterious effect of the novel βN548W inactivating mutation, even in a super-active GC-1 background. These cells had no measurable luciferase activity at any time point (Fig. S6) and 92% lower extracellular cGMP levels compared with cells transformed with the αC595S/βM537N variant. Finally, cells expressing the triple αC595S/βM537N/βP538Q variant had high luciferase activity (7.0-fold greater than cells expressing WT GC-1), but 2-fold lower extracellular cGMP levels compared with cells expressing the double αC595S/βM537N GC-1 variant. This result suggested a dampening effect of the βP538Q mutation when combined with other activating mutations.

To test our previous hypothesis regarding an interfacial hydrogen-bond network among residues αCys-595, αGlu-526, and βThr-474 (13), we made the βT474V, the αC595S/αE526A, and the αC595S/βT474V variants (Fig. 1*D*). Surprisingly, cells expressing the βT474V variant had luciferase activity and cGMP levels 4.6- and 2.1-fold higher than cells expressing WT GC-1, respectively (Table 1). This result suggested that removal of the hydroxyl group was activating, contrary to what was expected if this residue participated in a hydrogen-bond network. To test whether hydrophobic interactions at the dimer interface may be beneficial for activity, we made the βT474M GC-1 variant. Cells transformed with this variant had increased luciferase activity and extracellular cGMP levels compared with the βT474V variant. In addition, the double αC595S/αE526A and αC595S/βT474V variants showed increased luciferase and cGMP activity compared with WT GC-1. These results challenged the previous hydrogen-bonding network hypothesis, and suggested instead that increasing hydrophobic interactions at the dimer interface may constitute a novel mechanism for activating GC-1.

### βC541G mutation reduces substrate specificity and synergizes with other variants to activate GC-1

The βCys-541 residue was proposed to play a key role in substrate specificity (4, 13, 38, 39) and is conserved in guanylyl cyclases (Figs. 1*E* and 2). Cells expressing the βC541G variant had luciferase activity and cGMP levels 6.6- and 2.2-fold greater than cells expressing WT GC-1, respectively. In addition, they had extracellular cAMP levels 7.6-fold higher than cells expressing WT GC-1 (Table 1). As a consequence, βC541G GC-1 showed drastically reduced substrate specificity compared with WT GC-1 (cGMP/cAMP ratio of 0.9 and 3, respectively).

To identify potential synergy between multiple mutations, we combined the substrate-binding cleft βC541G mutation with activating mutations at the dimer interface and in the dorsal flaps. Cells expressing the double αC595S/βC541G variant displayed luciferase activity and cGMP levels 7.5- and 13.2-fold greater than cells expressing WT GC-1, respectively. Cells expressing the triple αC595S/βC541G/βM537N variant had luciferase activity and cGMP levels 7.1- and 15.3-fold higher than cells expressing WT GC-1 (Fig. S7), making it the highest-activity variant so far. Cells expressing either the αC595S/βC541G or αC595S/βM537N/βC541G variants also displayed increased extracellular cAMP levels compared with WT GC-1 (6.2- and 5.3-fold, respectively). These cells had substrate specificity for GTP exceeding that of WT GC-1, despite the βC541G mutation. Finally, cells expressing the quadruple αC595S/βM537N/βP538Q/βC541G variant displayed luciferase activity and cGMP levels 1.1- and 5.3-fold greater than cells expressing WT GC-1. This phenotype confirmed a dampening effect induced by the βP538Q mutation when combined with other activating mutations.

### Activating mutations cause a global subunit rotation leading to enhanced ventral inter-subunit coupling and rearranged dorsal inter-subunit coupling

**Figure 3.**
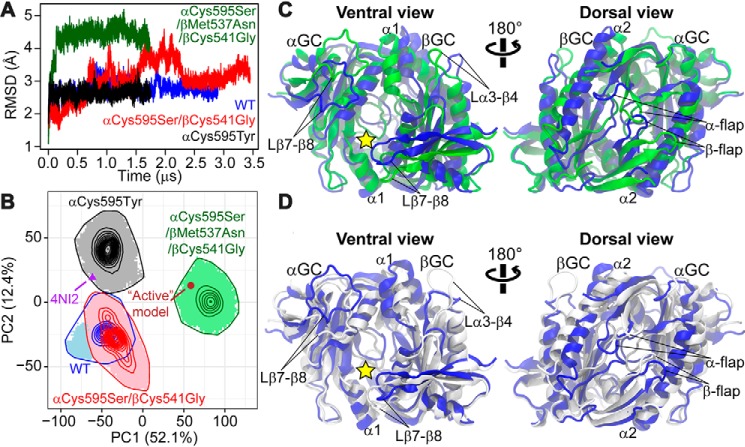
**Molecular dynamics simulations reveal distinct backbone conformations among WT and mutant αβGC^cat^ that are related to GC-1 activation.** *A,* RMSD of backbone atoms, with respect to the crystallographic structure (PDB code 4NI2), derived from MD simulations for WT and αβGC^cat^ mutants. *B,* PCA performed on Cartesian coordinates of backbone atoms from the simulations. Simulation-generated conformational snapshots are projected as *shaded areas* in the subspace spanned by the two principal components capturing the largest structural variance (PC1 and PC2; the number in the axis label indicates the percentage of variance captured by the corresponding PC). *Contour lines* represent probability density distributions of conformational samples, where the outmost line indicates the boundary of sampled space. The crystallographic structure representing the inactive conformation (PDB code 4NI2) and the active structural model are also mapped. *C* and *D,* collective motions represented by PC1 and PC2, respectively. Two extreme interpolated structures of αβGC^cat^ along each PC are superimposed and represented as cartoons. *C, blue* and *green* are structures at −100 and 120, respectively, along PC1. *D, blue* and *white* represent −70 and 70, respectively, along PC2. The substrate-binding site is indicated by a *yellow star*.

To obtain structural insights into sGC activation, we used MD simulations. We performed four long-time (1.7–3.5 μs) MD simulations for the heterodimeric catalytic domains of WT GC-1, two activating mutants (αC595S/βC541G and αC595S/βM537N/βC541G), and a mutant with impaired GTP cyclase activity (αC595Y). All simulations started from the crystallographic structure representing an inactive conformation (13) and were carried out under the ligand-bound condition (Mg^2+^ and GTP). After simulations, the two activating mutants reached conformations that were farther away from the initial inactive conformation than the deactivating mutant (Fig. 3*A*). In particular, the activating triple mutant quickly reached a backbone root mean square deviation (RMSD) above 4 Å within 100 ns, whereas the low-activity mutant retained conformations with backbone RMSD <3 Å throughout the simulation. This indicated that whereas the αC595Y mutation indeed stabilized the enzyme in the initial inactive state, activating mutations caused a shift of free energy landscape for the system to explore new conformations.

The distinct backbone conformations in equilibrium identified by MD for WT and mutant αβGC^cat^ are relevant to sGC activation. We used principal component analysis (PCA) to compare conformational ensembles generated by MD simulations. Only the equilibrated part of each simulation was considered. Projections of simulations in the subspace spanned by the top two principal components (*i.e.* PC1 and PC2), which collectively captured nearly 65% of total structural variance, showed that the activating triple mutant was separated from WT αβGC^cat^ mainly by PC1, whereas the low-activity αC595Y mutant deviated from WT αβGC^cat^ along PC2 (Fig. 3*B*). These results suggested that PC1 was relevant to activation, whereas PC2 was relevant to inhibition. Although the activating double mutant overlapped with WT αβGC^cat^, it sampled a unique conformational space that was farther away from the deactivating mutant and closer to the activating triple mutant (Fig. 3*B*). Intriguingly, conformations populated by the activating triple mutant resembled the previous “active” structural model of αβGC^cat^ (13), as shown by the projection of the active model in the PC1–PC2 subspace, which fell in the region sampled exclusively by the triple mutant. Importantly, this result corroborated the experimental observation that the triple mutant acquired dramatically enhanced catalytic activity (see above). In contrast, the projection of the inactive crystallographic structure was located in the region sampled only by the deactivating mutant, supporting experimental results showing that the mutation caused a substantial decrease in measured extracellular cGMP levels.

Our results suggested that, upon mutation, GC-1 activation involved a global subunit rotation along with local conformational rearrangements including changes along the dorsal flaps. The collective motion represented by PC1 contained an obvious relative rotation of the two subunits (Fig. 3*C* and Movie S1). On the ventral side of the enzyme, the subunit rotation resulted in a conformation where the loop between the β7 and β8 strands (Lβ7–β8, residues 639–653) in the α (or β, residues 591–598) subunit was closer to the α1 helix (residues 430–437) in the β (or α, residues 490–496) subunit, potentially enhancing interactions in these regions. The movement of the Lβ7–β8 loop in the β subunit may also more widely expose the hypothetical pseudo-symmetric pocket, located next to the active-site pocket and a potential binding site for allosteric modulators (4). On the dorsal side, an interesting asymmetric conformational rearrangement around the flaps was observed during the transition along PC1. Although the β-flap moved toward the α2 helix of the α subunit (residues 499–520), the α-flap moved away from the β-subunit α2 helix (residues 443–461).

On the other hand, the collective motion represented by PC2 mainly contained local conformational rearrangements in both ventral and dorsal surfaces (Fig. 3*D* and Movie S2). On the ventral side, we observed that the Lβ7–β8 loops from both subunits were further separated from the α1 helix in the adjacent subunit in the deactivating mutant compared with WT αβGC^cat^. On the dorsal side, a similar detachment from the enzyme was observed for the dorsal α-flap, whereas the dorsal β-flap slightly twisted.

**Figure 4.**
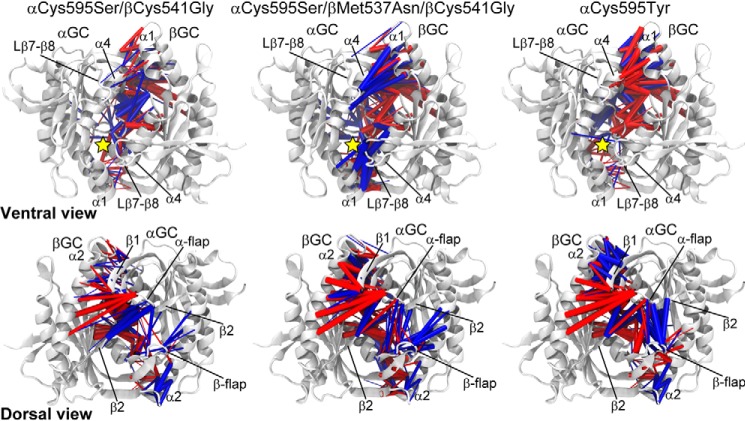
**Function-related rearrangements of interfacial residue-residue contacts/interactions upon mutation.** Contact probability changes (denoted by *df*) from WT αβGC^cat^ to a specific mutant (column) are mapped to the crystallographic structure of αβGC^cat^ (PDB code 4NI2, *white cartoon*). Only contacts between subunits are shown for clarity. *Blue* and *red cylinders* represent contacts with df ≥ 0.1 and df ≤−0.1, respectively, where the *cylinder* radius is proportional to |df|. The substrate-binding site is indicated by a *yellow star*.

We showed that conformational changes during GC-1 activation led to enhanced inter-subunit residue-residue interactions on the ventral surface and substantial interaction rearrangements on the dorsal surface. Residue-residue contacts were examined for each simulation using our recently developed method (40). Briefly, the probability that two residues form a contact during a simulation was calculated for each mutant and compared with the corresponding probability calculated for WT αβGC^cat^. Significant changes in contact probability (>0.1) were then mapped to the molecular structure of αβGC^cat^ for visual inspection. We showed that in the triple mutant, contacts were more often formed on the ventral side of the enzyme (Fig. 4). These included contacts between the α-subunit Lβ7–β8 loop and the β-subunit α1 helix, the α-subunit α4 helix (599–610) and the β-subunit α1 helix, and their symmetric counterparts. In contrast, in the low-activity mutant, contacts in these regions showed an either similar or substantially reduced formation probability with respect to WT αβGC^cat^, suggesting decoupling of these structural segments. The dorsal α-flap lost contacts with the β-subunit α2 helix in both the activating triple mutant and the deactivating mutant. However, the β1 strand, neighboring the strands forming the dorsal α-flap, more frequently formed contacts with β-subunit α2 helix in the deactivating mutant. Similarly, although the dorsal β-flap formed more contacts with the α subunit in both the activating triple mutant and the deactivating mutant, their pattern of contact formation differed and resulted in significantly rearranged residue-residue interactions from the inactive to the active state. In the activating triple mutant, the β-flap made more contacts with the α-subunit α2 helix, whereas in the αC595Y mutant, it detached from the α-subunit α2 helix and formed stronger contacts with the α-flap and the α-subunit β2 strand. Key contact changes in the activating double mutant were overall similar to those in the activating triple mutant, although their magnitudes were smaller in the double mutant. This was observed for both residue-focused difference-contact networks (Fig. 4) and coarse-grained networks displaying net contact changes between residue segments (Figs. S8 and S9).

## Discussion

The mechanism by which NO-binding may promote a series of activating conformational changes from the N-terminal HNOX domain to the C-terminal cyclase domain is unknown. To fill the gap in our understanding of the activation mechanism at the molecular level, we developed a novel assay that reports on heterologous GC-1 activity in bacterial cells. To our knowledge, this is the first time that a bacterial reporter assay was used to identify mutations in sGC that affect its activity, and thus represents a significant advance in the field that will likely accelerate structure-function studies. The assay, which is faster and cheaper than available cAMP/cGMP immunoassays, allowed rapid identification of mutants associated with apparent increased GC-1 activity. Follow-up studies with immunoassays then allowed us to differentiate between various GC-1 nucleotidyl cyclase activities. For most mutants, there was good agreement between luciferase and cGMP increases compared with WT GC-1. However, discrepancies occurred for mutants with increased cAMP synthesis activity, confirming the promiscuity of the luciferase promoter. Thus our assay allowed for the first time identification of mutations that affected both cAMP and cGMP production, confirming that sGC has broader substrate specificity than originally thought (41). Although we did not do these, cyclic nucleotide-specific immunoassays could subsequently be used to further determine the catalytic parameters of interesting mutants.

We and others have defined the key structural elements of the cyclase domain as the dorsal flaps, dimer interface, and substrate-binding region (12, 13, 27, 28, 39, 42). We used our assay to systematically define the role of these structural elements in the activation mechanism.

The exact role of the dorsal flaps in sGC activation is unknown, however, one of the only two activating mutations previously known (βM537N) belongs to the βGC^cat^ dorsal flap (28). Importantly, our luciferase assay and extracellular cGMP measurements confirmed that the βM537N mutation was activating and further validated the assay for identification of gain-of-function GC-1 variants. In addition, we identified novel mutations in both flaps (βI533M, βP538Q, αM591N, the double αV587I/V589T and triple αV587I/V589T/K590R), and the replacement of both flaps with Ala-Gly dipeptides, which all yielded increased GC-1 activity.

We previously hypothesized that this structural element was a key modulator of the orientation of the catalytic subunits necessary for catalytic activity (13). We showed that the dorsal flaps were highly conserved between sGC and AC enzymes, and contained the consensus sequence Gly-Val-Ile-Gly-X_5_-Tyr (Fig. 2), except for the C2 subunit from *Ovis aries* AC-IV, which lacked a dorsal flap altogether, and that of *Mycobacterium tuberculosis* AC, which only contained an Arg-Ala-Gly tripeptide (16). Interestingly, the flaps adopted asymmetric conformations in several catalytic domain crystal structures, including our X-ray structure of the sGC heterodimeric catalytic domains (13) and structures of AC catalytic domains or full-length enzyme (17–19, 43, 44). In structures that included the preceding coiled-coil domain, one flap (equivalent to the βGC^cat^ flap) packed snuggly onto the adjacent catalytic subunit, whereas the other (equivalent to the αGC^cat^ flap) packed closer to the adjacent helix-turn-helix motif of the coiled-coil domain. Interestingly, our independent MD simulations also showed an asymmetric conformation of the flaps in mutant αβGC^cat^ (see above). Such asymmetric arrangement may allow tight coupling of the catalytic subunits with the coiled-coil domain thought to transmit the activation signal to the active site (45–47).

**Figure 5.**
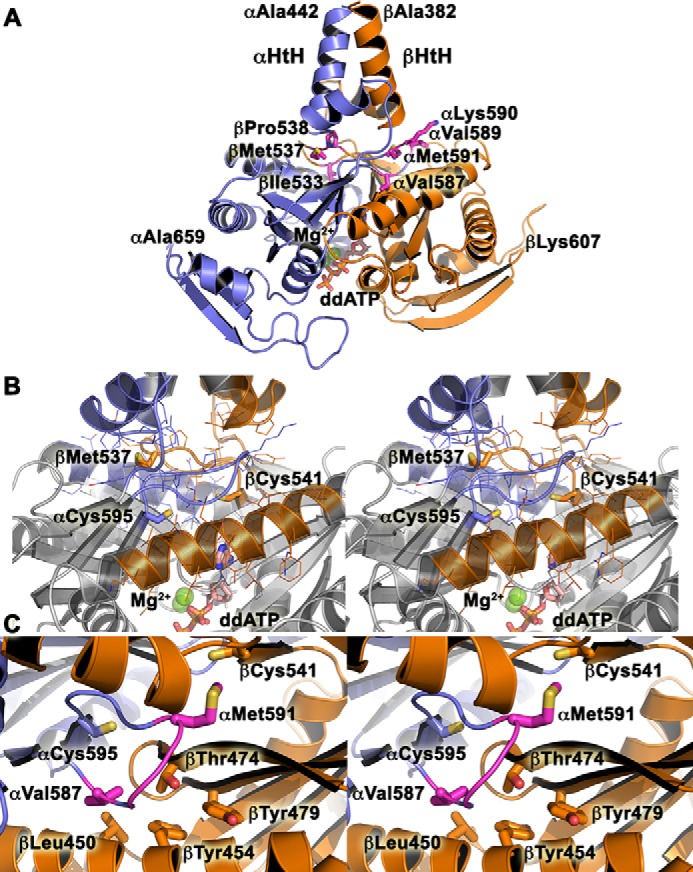
**Model for WT CC-GC^cat^.** *A,* the model for αGC-1 (442–659, *blue*) and βGC-1 (383–607, *orange*) was generated with SWISSMODEL (77, 78). Key residues from the dorsal flaps mutated in this study are shown as *sticks* and labeled. The helix-turn-helix (α*HtH* and β*HtH*) motifs of the penultimate coiled-coil domain are indicated. Magnesium ions (*green balls*) and dideoxy-ATP (*sticks*, ddATP) are included in the model. *B,* stereoview of the central positioning for the dorsal flaps, which are sandwiched between the helix-turn-helix motif and the active site of the catalytic domain. The view is the same as in *A*. Key residues are shown in *sticks* and colored in *blue* (αGC) and *orange* (βGC). *C,* stereoview of the hydrophobic pocket around residue βThr-474.

To understand the potential effect of the flap mutations, we generated a model for residues β382–607 and α442–659 based on the homologous Cya_SOL_ structure (PDB code 5O5K; sequence identity between Cya_SOL_ residues 204–436 and sGC is 33% for α442–659 and 36% for β382–607), which contains the helix-turn-helix motif at the end of the coiled-coil domain (48). The resulting model (Fig. 5*A*) was compatible with previous studies (24–26) and the recent 4 Å resolution cryo-EM structure of full-length sGC (47). In particular, it superimposed better to the activated state than to the inactive state of full-length sGC (PDB code 6JT2; RMSD = 1.6 Å and PDB code 6JT0; RMSD = 2.3 Å for 371 residues, respectively). In this model, the dorsal flaps are centrally located to connect the coiled-coil domain to the active site. Residues βIle-533 and αVal-587 are located at the interface with the adjacent catalytic subunit and their mutation into larger hydrophobic amino acids may stabilize the nearby hydrophobic pocket, as proposed previously (37). In contrast, βPro-538 and αLys-590 are located at the interface with the adjacent coiled-coil domain, and their mutation could alter interactions between these structural elements. Finally, residues βMet-537, αVal-589, and αMet-591 are all sandwiched between the catalytic and coiled-coil domains, and their mutations into hydrogen-bonding amino acids could modify the interface between these domains as well-as the active site conformation (Fig. 5*B*). Overall, our results show that altering the balance of interactions between the dorsal flaps and the coiled-coil domain on one side, and the dorsal flaps and the catalytic domains on the other side, either by mutation or deletion of the flaps leads to sGC activation. This strongly confirms a key allosteric regulatory role for the dorsal flaps in transmitting conformational changes from the coiled-coil domain to the catalytic center via the helix-turn-helix motif, which was shown to be involved in this signal transduction cascade in sGC and AC (17, 19, 21, 25, 43, 46, 49).

Importantly, our studies identify for the first time synergy between the dorsal flaps, the dimer interface, and the substrate-binding region of the catalytic domain to increase sGC activity. We identified a network a hot spots that not only activates sGC but also affects its substrate specificity.

The intersubunit αC595S mutation was the second activating mutation ever identified for sGC (27, 50). Accordingly, we measured the highest extracellular cGMP levels in cells transformed with this variant out of all single-point mutations measured in this study. Our current results with the novel βT474V, βT474M, αC595S/βT474V, and αC595S/αE526A variants showed that these residues are not involved in an interfacial hydrogen-bonding network, as previously proposed (13). The βThr-474 residue is located in a hydrophobic pocket lined with residues βLeu-450, βTyr-454, βTyr-479, αPhe-597, and dorsal flap residue αVal-587 (Fig. 5*C*), whose mutation into a larger hydrophobic residue also increased GC-1 activity. Based on our results, we propose that the βT474V, βT474M, and αV587I mutations fill a hydrophobic pocket at the interface between the helix-turn-helix motif of the coiled-coil domain and the active site to promote sGC activation. Our results thus point to an intricate coupling between various structural elements in the catalytic domains, a mechanism that has not yet been described for other cyclases. This is further confirmed by the synergistic increase in activity observed for the double αC595S/βM537N variant. These residues are located 8 Å apart and link the coiled-coil domain to the active site center via the dorsal flap. Their mutation into hydrogen-bonding residues could therefore alter the interface between the coiled-coil domain and the substrate-binding regions, leading to an active conformation. We propose that αCys-595, βThr-474, and βMet-537 are part of a network of amino acids that connect the coiled-coil domain to the active site. This network of amino acids extends all the way to the substrate-binding regions, as shown by our results with the βC541G GC-1 variant.

**Figure 6.**
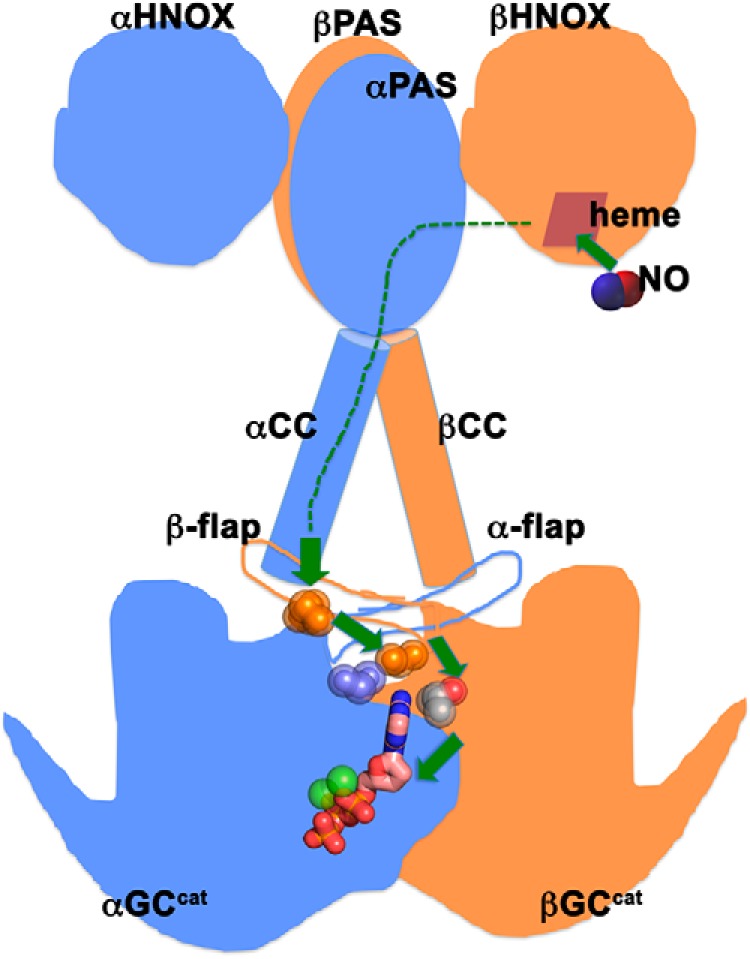
**Proposed mechanism for sGC activation.** Schematic representation of full-length sGC with the N-terminal HNOX domains, the dimerization domains (*PAS*), the coiled-coil domains (*CC*), and the C-terminal catalytic domains (*GC^cat^*). Upon NO binding to the HNOX heme, the activation signal gets transmitted through all the domains (*dashed green lines*). Hot spot residues (*spheres*) belonging to coupled networks in the dorsal flaps, the intersubunit interface, and the active site couple the preceding CC domains to the GC^cat^ domains to promote activation.

The βCys-541 residue is characteristic of all GCs, which possess a Glu-Cys pair thought to be involved in nucleobase specificity via hydrogen bonds (51), except in *Synechocystis PCC6803* GC (GC_cya2_), which shows even greater specificity for GTP (38) and where the Cys residue is replaced by Gly-562. Several βCys-541 mutations have been shown to influence GC-1 substrate specificity, activity, and response to NO (27, 39). Accordingly, we predicted that the βC541G variant would be highly specific for GTP over ATP. Surprisingly, this variant displayed greater activity than WT GC-1 but lost GTP substrate specificity. Additionally, combination of this mutation with activating mutations at the dimer interface (αC595S/βC541G) and the dorsal flap (αC595S/βM537N/βC541G) resulted in a synergistic increase in activity and increased GTP specificity compared with WT GC-1. Our results thus suggest that the role of βCys-541 is more complex than simply providing interaction to the nucleobase. Instead, we propose that αCys-595, βThr-474, βMet-537, and βCys-541 are part of a network of hot spots that allow transmission of the NO signaling events from the HNOX domain through the coiled-coil domain to the active center of the catalytic domain (Fig. 6).

Not all combinations of mutations were found to be synergistic. In particular, the βP538Q mutation was found to dampen the synergistic activation observed for other mutants described above. Although the single-point variant had a modest increase in GC-1 activity, similar to the AC P1015Q mutation (37), the quadruple αC595S/βM537N/βC541G/βP538Q variant had about half the activity of the highly active αC595S/βM537N/βC541G variant. This was also true for the αC595S/βM537N/βP538Q *versus* αC595S/βM537N variants, and the αC595S/βP538Q *versus* the αC595S variants. These results corroborate previous results that suggested that Pro-1015 was highly influential on AC activity (52). The dorsal flap βPro-538 residue is located at the interface with the coiled-coil domain preceding the catalytic domain (Fig. 5*B*). In all the multiple variants cited above, mutation of the rigid βPro-538 into a more flexible hydrogen-bonding residue had a negative effect on activity. Our results thus confirm our hypothesis that a fine balance of interactions mediated by the dorsal flaps with the coiled-coil domain and the catalytic domain is critical to sGC activity.

With MD and associated statistical analyses, we obtained high-resolution atomic insights into activating and deactivating mechanisms of GC-1 variants. First, MD simulations identified that, in equilibrium, the synergistic activating triple αC595S/βM537N/βC541G mutant sampled conformations that resembled the modeled active structure, whereas the deactivating αC595Y mutant stayed around the initial inactive conformation defined crystallographically, validating the predictive power of our simulations. A subsequent comparative study of conformational ensembles under distinct mutational conditions revealed both backbone conformational changes and side chain rearrangements (represented by residue-residue contacts) relevant to GC-1 activation/inhibition. Surprisingly, the activating double αC595S/βC541G mutant sampled conformations similarly to WT αβGC^cat^. However, the trends of conformational changes between the double mutant and WT αβGC^cat^ were similar to those observed for the triple mutant, although the magnitude of changes were smaller in the former. This mirrored the experimental results showing a smaller increase in extracellular cGMP levels for the double mutant compared with the triple mutant. Our computational findings revealed additional hot spots (*e.g.* residues in the ventral α1 and α4 helices, and the Lβ7–β8 loop) that can be harnessed to modulate GC-1 activities. This knowledge may be further leveraged to design new mutants and develop novel allosteric drugs targeting GC-1.

In summary, we optimized a luciferase reporter assay for sGC activity and identified several activating variants in the catalytic domains that work in concert to coordinate GTP cyclase activity. We showed that some of these residues affected not only the GTP cyclase activity but also its substrate specificity. This is the first time that synergy is observed among various regions of the sGC catalytic domains, a mechanism that has never been described in other nucleotidyl cyclases. Based on our results, we propose that residues in these regions constitute hot spot linkages in the sGC NO-induced activation cascade to propagate the activating signal through the penultimate coiled-coil domain to the active site. In particular, the dorsal flaps act as an interfacial switch between the helix-turn-helix motif and the catalytic domains, a mechanism likely shared with other class III nucleotide cyclases. Our molecular dynamics studies supported the key role of the dorsal flaps in sGC activation and highlighted additional interfacial regions impacted by activating mutations. Importantly, our results further our understanding of the mechanisms by which sGC activity is regulated and provide new routes for rational design of small molecules that target these allosteric regions to improve dysfunctional sGC activity in cardiovascular diseases. The described luciferase reporter system will be used as an unbiased screening tool to identify additional mutations that activate sGC and/or alter its substrate specificity via random mutagenesis. It is therefore an exciting new tool to understand the evolutionary relationships between guanylyl cyclases and related adenylyl cyclases, and understand how these enzymes may have evolved distinct activation mechanisms.

## Experimental procedures

### Materials

Antibiotics were used at the following final concentrations: ampicillin (Amp), 35 μg/ml; kanamycin (Kan), 50 μg/ml; chloramphenicol (Chlor), 35 μg/ml; and spectinomycin (Spect), 50 μg/ml.

### Plasmids and gene construction

Codon-optimized human αGC-1 (residues 1–690) and βGC-1 (residues 1–619) DNAs were purchased from BioBasic. The αGC-1 polypeptide with an N-terminal His_6_-thioredoxin-His_6_-SUMO tag was cloned into pCDF-Duet (Spect^r^, Novagen) MCS-1 between NcoI and EcoRI sites; the βGC-1 polypeptide with an N-terminal monomeric OCR (Mocr) (53) tag was cloned into pCDF-Duet MCS-2 between NdeI and XhoI sites. Because the BL21(DE3) *cyaA*^−^ cells were also spectinomycin resistant, we replaced the Spect^r^ gene of the pCDF-Duet vector with the Amp^r^ gene from the pET-21a vector (supporting data). The final plasmid, which contained both GC-1 polypeptide chains, was denoted pCDF-αβGC1 (Amp^r^). We also generated the pCDF-αGC1 (Amp^r^) plasmid, which only contained the αGC-1 polypeptide in MCS-1 as a negative control. Mutations were introduced using site-directed mutagenesis (Agilent). All sequences were confirmed by sequencing (GENEWIZ).

### Cell growth conditions

To reproducibly obtain cell growth, BL21(DE3) cells (Life Technologies) and BL21(DE3) *cyaA*^−^ cells (Spect^r^) were first transformed with pOPTXcGMPRE:LUC (Kan^r^), plated on LB agar plates with antibiotics, and made chemically-competent. We then co-transformed these cells with the compatible vectors pGro7 (Chlr^r^, Takara Inc.) and pCDF-αβGC1 or pCDF-αGC1 (Amp^r^) and plated on LB agar plates with antibiotics (Table S1). Overnight cultures grew at 37 °C, 225 RPM in Luria Broth (LB). The next day, 100 ml of Terrific Broth (TB) medium were inoculated with 3 ml of overnight culture and grown at 37 °C, 225 rpm until *A*_600_ reached ∼0.2–0.4. Cells were put on ice for 1 h and induced with 100 μm isopropyl β-d-1-thiogalactopyranoside, 0.2% (w/v) arabinose, 0.45 mm δ-aminolevulinic acid, and 30 μm ferric citrate. Induced cultures grew at 15 °C, 90 RPM for up to 72 h. Samples (1.5 ml) were pelleted at various time points, and stored at −80 °C until further use.

### Western blotting sample preparation

We resuspended 1.5-ml cell pellets in a volume of SDS-PAGE loading buffer normalized to the *A*_600_. Samples were incubated at 70 °C for 8 min and centrifuged before loading onto a 13% acrylamide gel (5 μl of sample/lane).

### Luciferase assay

Cell lysates for the luciferase assay were prepared following the Promega protocol with slight modifications. The 1× cell culture lysis reagent was supplemented with 50 units/ml of benzonase. To maximize the signal from luciferase activity in the cell lysate, we optimized the volume of cells that were harvested, as well-as the volume of lysis buffer for resuspension. We resuspended 1.5-ml pellets in 100 μl of 100 mm K_2_HPO_4_ (pH 7.4) and 2 mm EDTA. Each sample received 300 μl of 1× cell culture lysis reagent. Lysis was accomplished via mechanical disruption with glass beads and clarified at 10,000 × *g* for 5 min at 4 °C.

Luciferase activity was measured in clarified cell lysates using a GloMax Multi+ Microplate Multimode Reader. We transferred 20 μl of clarified lysate into a 96-well-black bottom plate, added 100 μl of luciferin substrate (Promega) to each well, and the signal was integrated over 10 s with a 2-s delay between wells. All data were normalized to protein concentrations measured using the bicinchoninic acid assay (Pierce). Background luciferase activity was measured from cells co-transformed with pOPTXcGMPRE:LUC, pGro7, and pCDF-αGC1 lacking the βGC-1 polypeptide (Fig. S2).

### cGMP/cAMP levels with a competitive ELISA

Extracellular and intracellular cGMP and cAMP levels were measured using the Parameter Assay Kits (R&D Systems) following the manufacturer's protocol. We washed 1.5-ml cell pellets once in PBS and resuspended them in 280 μl of lysis buffer (Cell Lysis Buffer-5, 1 mg/ml of lysozyme, 25 units/ml of benzonase, and 0.3 mm 3-isobutyl-1-methylxanthine). Lysis was accomplished with glass beads and clarified at 4,000 × *g* for 10 min at 4 °C. Samples were stored at −80 °C until further use. Background extracellular cGMP levels were measured in TB medium. Background extracellular cAMP levels were measured from BL21(DE3) *cyaA*^−^ cells co-transformed with pCDF-αGC1, pOPTXcGMPRE:LUC, and pGro7, and grown at 15 °C, 90 RPM for 72 h. All extracellular cyclic nucleotide concentrations were normalized to cell lysate protein concentrations measured using the bicinchonic acid assay (Pierce). The cGMP/cAMP signal was measured using a SpectraMax plate reader.

### Molecular dynamics simulation

MD simulations were performed using AMBER16 (54) along with the AMBER ff14SB force-field (55, 56). The apo inactive crystallographic structure was used as the starting conformation for all simulations (13). Initial coordinates for the ligands (Mg^2+^, GTP) were taken from the previously modeled active structure (13) after a least square structural superimposition based on backbone atoms. Force-field parameters for the nucleotide were taken from Meagher *et al.* (57). The protonation state of histidine was determined based on the p*K_a_* value at pH 7.0 calculated by PROPKA 3.0 (58) implemented in PDB2PQR 2.1 (59), along with a visual inspection of their structural environment. Mutations were introduced by first removing side chain atoms of mutational sites and changing their residue names. Missing atoms were then added by AMBER16. Each system was solvated in an octahedron box filled with pre-equilibrated TIP3P water molecules (60), which extended 10 Å from the surface of solute to each box face. Original water molecules in the crystallographic structure were kept. Na^+^ or Cl^−^ counterions were added to neutralize systems. Energy minimization was performed with 2,000 steps of steepest decent followed by 3,000 steps of conjugate gradient, where the position of solute was held by harmonic restraint. Five rounds of energy minimization were performed, where the force constant of the positional restraint was gradually reduced from 500 kcal mol^−1^ Å^−2^ to 0. Each system was then heated up from 100 to 300 K within 500 ps under NVT periodic conditions, with a 1-fs time step where solute was held in the same way as in the energy minimization procedure. Five rounds of heating were performed, where the force constant of restraint was set to 500, 300, 100, 50, and 5 (kcal mol^−1^ Å^−2^). A 1-ns equilibration was performed with a 2-fs time step and no restraint under NPT (300 K, 1 bar) periodic conditions. Subsequent production of MD was then performed under the same conditions as equilibration for 1.7–3.5 μs. The last 1.0–1.5 μs of production was considered as equilibrated, during which the backbone conformation of the system was stabilized except for small thermal fluctuations. The temperature was coupled to a Langevin thermostat with collision frequency γ = 1.0 ps^−1^ and pressure coupled to a Monte Carlo barostat with coupling constant τ_p_ = 1.0 ps. The particle-mesh Ewald summation method (61) was employed to treat long-range electrostatic interactions. For short-range nonbonded interactions, a 9-Å cutoff was used. All bonds involving hydrogen atoms were constrained with the SHAKE algorithm (62). Trajectories were saved every 1 ps.

### PCA

PCA was applied to the MD simulations using the CPPTRAJ command (63) of AMBER16. Prior to PCA, structural superimpositions were performed based on backbone atoms for all simulated conformational snapshots. The variance-covariance matrix characterizing correlated internal backbone motions was then calculated and diagonalized to obtain the eigenvectors or principal components (PCs). The structural variance along each PC is given by the corresponding eigenvalue. The first two PCs capturing the largest structural variance (PC1 and PC2) were used to build the subspace where simulation trajectories were projected for the subsequent inter-conformer relationship analysis. The plot of PC1-PC2 projections was generated by R 3.5 and ggplot2 3.1 (64). Molecular graphics were generated by VMD 1.9 (65). Movies were made by VMD 1.9 and Photoshop CC (Adobe Inc.).

### Difference contact network analysis

The difference contact network analysis (dCNA) method previously reported (40) was employed. Briefly, a contact is formed if any pair of heavy atoms between two residues are within a distance of 4.5 Å, as previously used (40, 66, 67). The probability of contact formation for each residue pair during a simulation was then calculated. Contact probability changes, df, from WT αβGC^cat^ to each mutant were mapped to the molecular structure of αβGC^cat^. Contact probability differences were considered significant if their absolute values were greater than the estimated statistical error (0.1) (66) and the two residues were at least three amino acid apart (*i* to *i* + *n*, *n* ≥ 3). Residue-residue contacts were further used to identify intrinsic modular structures, or communities, of αβGC^cat^ as previously described (40), which in turn summarized residue wise contact changes by calculating net contact changes between communities. The network analysis and associated graphics generation were performed with bio3d 2.3 (68, 69) and igraph 1.2 (70).

## Author contributions

K. C. C., X.-Q. Y., D. H., and E. D. G. conceptualization; K. C. C., X.-Q. Y., S. G., J. A., D. H., and E. D. G. formal analysis; K. C. C., D. H., and E. D. G. supervision; K. C. C., D. H., and E. D. G. funding acquisition; K. C. C., X.-Q. Y., S. G., and E. D. G. validation; K. C. C., X.-Q. Y., S. G., J. A., D. H., and E. D. G. investigation; K. C. C., X.-Q. Y., S. G., and E. D. G. visualization; K. C. C., X.-Q. Y., S. G., J. A., D. H., and E. D. G. methodology; K. C. C., X.-Q. Y., S. G., D. H., and E. D. G. writing-original draft; K. C. C., D. H., and E. D. G. project administration; K. C. C., X.-Q. Y., S. G., J. A., D. H., and E. D. G. writing-review and editing.

## Supplementary Material

Supporting Information

## Footnotes

5 The abbreviations used are:

sGC: soluble guanylyl cyclase
GC-1: α_1_β_1_ soluble guanylyl cyclase
AC: adenylyl cyclase
NO: nitric oxide
OPTXcGMPRE:LUC: oligopeptide transporter X cyclic guanosine 3′,5′-monophosphate reporter gene luciferase
MD: molecular dynamics
PCA: principal component analysis
RMSD: root-mean-square deviation
HNOX: heme-nitric oxide/oxygen
PC: principal component
RLU: relative light unit.

## References

[1] Ignarro L. J., and Freeman B. (eds) (2017) Nitric Oxide: Biology and Pathobiology, 3rd Ed., Academic Press, San Diego

[2] Montfort W. R., Wales J. A., and Weichsel A. (2017) Structure and activation of soluble guanylyl cyclase, the nitric oxide sensor. Antioxid. Redox Signal. 26, 107–121 10.1089/ars.2016.6693 26979942 PMC5240008

[3] Bian K., and Murad F. (2003) Nitric oxide (NO)–biogeneration, regulation, and relevance to human diseases. Front. Biosci. 8, d264–78 10.2741/997 12456375

[4] Childers K. C., and Garcin E. D. (2018) Structure/function of the soluble guanylyl cyclase catalytic domain. Nitric Oxide 77, 53–64 10.1016/j.niox.2018.04.008 29702251 PMC6005667

[5] Ma X., Sayed N., Beuve A., and van den Akker F. (2007) NO and CO differentially activate soluble guanylyl cyclase via a heme pivot-bend mechanism. EMBO J. 26, 578–588 10.1038/sj.emboj.7601521 17215864 PMC1783457

[6] Pellicena P., Karow D. S., Boon E. M., Marletta M. A., and Kuriyan J. (2004) Crystal structure of an oxygen-binding heme domain related to soluble guanylate cyclases. Proc. Natl. Acad. Sci. U.S.A. 101, 12854–12859 10.1073/pnas.0405188101 15326296 PMC516465

[7] Purohit R., Weichsel A., and Montfort W. R. (2013) Crystal structure of the Alpha subunit PAS domain from soluble guanylyl cyclase. Protein Sci. Publ. Protein Soc. 22, 1439–1444 10.1002/pro.2331 PMC3795502 23934793

[8] Ma X., Sayed N., Baskaran P., Beuve A., and van den Akker F. (2008) PAS-mediated dimerization of soluble guanylyl cyclase revealed by signal transduction histidine kinase domain crystal structure. J. Biol. Chem. 283, 1167–1178 10.1074/jbc.M706218200 18006497 PMC3010369

[9] Ma X., Beuve A., and van den Akker F. (2010) Crystal structure of the signaling helix coiled-coil domain of the β1 subunit of the soluble guanylyl cyclase. BMC Struct. Biol. 10, 2 10.1186/1472-6807-10-2 20105301 PMC2828450

[10] Nioche P., Berka V., Vipond J., Minton N., Tsai A. L., and Raman C. S. (2004) Femtomolar sensitivity of a NO sensor from *Clostridium botulinum*. Science 306, 1550–1553 10.1126/science.1103596 15472039

[11] Martin F., Baskaran P., Ma X., Dunten P. W., Schaefer M., Stasch J.-P., Beuve A., and van den Akker F. (2010) Structure of cinaciguat (BAY 58–2667) bound to Nostoc H-NOX domain reveals insights into heme-mimetic activation of the soluble guanylyl cyclase. J. Biol. Chem. 285, 22651–22657 10.1074/jbc.M110.111559 20463019 PMC2903410

[12] Allerston C. K., von Delft F., and Gileadi O. (2013) Crystal structures of the catalytic domain of human soluble guanylate cyclase. PloS One 8, e57644 10.1371/journal.pone.0057644 23505436 PMC3591389

[13] Seeger F., Quintyn R., Tanimoto A., Williams G. J., Tainer J. A., Wysocki V. H., and Garcin E. D. (2014) Interfacial residues promote an optimal alignment of the catalytic center in human soluble guanylate cyclase: heterodimerization is required but not sufficient for activity. Biochemistry 53, 2153–2165 10.1021/bi500129k 24669844 PMC3985721

[14] Tesmer J. J., Sunahara R. K., Johnson R. A., Gosselin G., Gilman A. G., and Sprang S. R. (1999) Two-metal-ion catalysis in adenylyl cyclase. Science 285, 756–760 10.1126/science.285.5428.756 10427002

[15] Tesmer J. J., Sunahara R. K., Gilman A. G., and Sprang S. R. (1997) Crystal structure of the catalytic domains of adenylyl cyclase in a complex with Gs·GTPS. Science 278, 1907–1916 10.1126/science.278.5345.1907 9417641

[16] Tews I., Findeisen F., Sinning I., Schultz A., Schultz J. E., and Linder J. U. (2005) The structure of a pH-sensing mycobacterial adenylyl cyclase holoenzyme. Science 308, 1020–1023 10.1126/science.1107642 15890882

[17] Vercellino I., Rezabkova L., Olieric V., Polyhach Y., Weinert T., Kammerer R. A., Jeschke G., and Korkhov V. M. (2017) Role of the nucleotidyl cyclase helical domain in catalytically active dimer formation. Proc. Natl. Acad. Sci. U.S.A. 114, E9821–E9828 10.1073/pnas.1712621114 29087332 PMC5699072

[18] Lindner R., Hartmann E., Tarnawski M., Winkler A., Frey D., Reinstein J., Meinhart A., and Schlichting I. (2017) Photoactivation mechanism of a bacterial light-regulated adenylyl cyclase. J. Mol. Biol. 429, 1336–1351 10.1016/j.jmb.2017.03.020 28336405

[19] Ohki M., Sugiyama K., Kawai F., Tanaka H., Nihei Y., Unzai S., Takebe M., Matsunaga S., Adachi S., Shibayama N., Zhou Z., Koyama R., Ikegaya Y., Takahashi T., Tame J. R., Iseki M., and Park S.-Y. (2016) Structural insight into photoactivation of an adenylate cyclase from a photosynthetic cyanobacterium. Proc. Natl. Acad. Sci. U.S.A. 113, 6659–6664 10.1073/pnas.1517520113 27247413 PMC4914150

[20] Mou T. C., Gille A., Suryanarayana S., Richter M., Seifert R., and Sprang S. R. (2006) Broad specificity of mammalian adenylyl cyclase for interaction with 2′,3′-substituted purine- and pyrimidine nucleotide inhibitors. Mol. Pharmacol. 70, 878–886 10.1124/mol.106.026427 16766715

[21] Steegborn C., Litvin T. N., Hess K. C., Capper A. B., Taussig R., Buck J., Levin L. R., and Wu H. (2005) A novel mechanism for adenylyl cyclase inhibition from the crystal structure of its complex with catechol estrogen. J. Biol. Chem. 280, 31754–31759 10.1074/jbc.M507144200 16002394 PMC3650720

[22] Saalau-Bethell S. M., Berdini V., Cleasby A., Congreve M., Coyle J. E., Lock V., Murray C. W., O'Brien M. A., Rich S. J., Sambrook T., Vinkovic M., Yon J. R., and Jhoti H. (2014) Crystal structure of human soluble adenylate cyclase reveals a distinct, highly flexible allosteric bicarbonate binding pocket. ChemMedChem 9, 823–832 10.1002/cmdc.201300480 24616449 PMC4506562

[23] Fritz B. G., Roberts S. A., Ahmed A., Breci L., Li W., Weichsel A., Brailey J. L., Wysocki V. H., Tama F., and Montfort W. R. (2013) Molecular model of a soluble guanylyl cyclase fragment determined by small-angle X-ray scattering and chemical cross-linking. Biochemistry 52, 1568–1582 10.1021/bi301570m 23363317 PMC3607398

[24] Underbakke E. S., Iavarone A. T., and Marletta M. A. (2013) Higher-order interactions bridge the nitric oxide receptor and catalytic domains of soluble guanylate cyclase. Proc. Natl. Acad. Sci. U.S.A. 110, 6777–6782 10.1073/pnas.1301934110 23572573 PMC3637750

[25] Underbakke E. S., Iavarone A. T., Chalmers M. J., Pascal B. D., Novick S., Griffin P. R., and Marletta M. A. (2014) Nitric oxide-induced conformational changes in soluble guanylate cyclase. Struct. Des. 22, 1–10 10.1016/j.str.2013.12.006 PMC4001857 24560804

[26] Campbell M. G., Underbakke E. S., Potter C. S., Carragher B., and Marletta M. A. (2014) Single-particle EM reveals the higher-order domain architecture of soluble guanylate cyclase. Proc. Natl. Acad. Sci. U.S.A. 111, 2960–2965 10.1073/pnas.1400711111 24516165 PMC3939929

[27] Friebe A., Russwurm M., Mergia E., and Koesling D. (1999) A Point-mutated guanylyl cyclase with features of the YC-1-stimulated enzyme: implications for the YC-1 binding site? Biochemistry 38, 15253–15257 10.1021/bi9908944 10563809

[28] Lamothe M., Chang F.-J., Balashova N., Shirokov R., and Beuve A. (2004) Functional characterization of nitric oxide and YC-1 activation of soluble guanylyl cyclase: structural implication for the YC-1 binding site? Biochemistry 43, 3039–3048 10.1021/bi0360051 15023055

[29] Wheeler J. I., Freihat L., and Irving H. R. (2013) A cyclic nucleotide sensitive promoter reporter system suitable for bacteria and plant cells. BMC Biotechnol. 13, 97 10.1186/1472-6750-13-97 24206622 PMC3829209

[30] Gomelsky M. (2011) cAMP, c-di-GMP, c-di-AMP and now cGMP: bacteria use them all! Mol. Microbiol. 79, 562–565 10.1111/j.1365-2958.2010.07514.x 21255104 PMC3079424

[31] Linder J. U. (2010) cGMP production in bacteria. Mol. Cell Biochem. 334, 215–219 10.1007/s11010-009-0321-0 19943185

[32] Ryu M. H., Moskvin O. V., Siltberg-Liberles J., and Gomelsky M. (2010) Natural and engineered photoactivated nucleotidyl cyclases for optogenetic applications. J. Biol. Chem. 285, 41501–41508 10.1074/jbc.M110.177600 21030591 PMC3009876

[33] Ryu M. H., Youn H., Kang I. H., and Gomelsky M. (2015) Identification of bacterial guanylate cyclases. Proteins Struct. Funct. Bioinform. 83, 799–804 10.1002/prot.24769 PMC4644428 25645367

[34] Datsenko K. A., and Wanner B. L. (2000) One-step inactivation of chromosomal genes in *Escherichia coli* K-12 using PCR products. Proc. Natl. Acad. Sci. U.S.A. 97, 6640–6645 10.1073/pnas.120163297 10829079 PMC18686

[35] Maathuis F. J. (2006) cGMP modulates gene transcription and cation transport in *Arabidopsis* roots. Plant J. 45, 700–711 10.1111/j.1365-313X.2005.02616.x 16460505

[36] Haase T., Haase N., Kraehling J. R., and Behrends S. (2010) Fluorescent fusion proteins of soluble guanylyl cyclase indicate proximity of the heme nitric oxide domain and catalytic domain. PloS One 5, e11617 10.1371/journal.pone.0011617 20657650 PMC2904703

[37] Hatley M. E., Benton B. K., Xu J., Manfredi J. P., Gilman A. G., and Sunahara R. K. (2000) Isolation and characterization of constitutively active mutants of mammalian adenylyl cyclase. J. Biol. Chem. 275, 38626–38632 10.1074/jbc.M007148200 10982815

[38] Rauch A., Leipelt M., Russwurm M., and Steegborn C. (2008) Crystal structure of the guanylyl cyclase Cya2. Proc. Natl. Acad. Sci. U.S.A. 105, 15720–15725 10.1073/pnas.0808473105 18840690 PMC2572937

[39] Sunahara R. K., Beuve A., Tesmer J. J., Sprang S. R., Garbers D. L., and Gilman A. G. (1998) Exchange of substrate and inhibitor specificities between adenylyl and guanylyl cyclases. J. Biol. Chem. 273, 16332–16338 10.1074/jbc.273.26.16332 9632695

[40] Yao X. Q., Momin M., and Hamelberg D. (2018) Elucidating allosteric communications in proteins with difference contact network analysis. J. Chem. Inf. Model. 58, 1325–1330 10.1021/acs.jcim.8b00250 29956925

[41] Beste K. Y., Burhenne H., Kaever V., Stasch J. P., and Seifert R. (2011) Nucleotidyl cyclase activity of soluble guanylyl cyclase α1β1. Biochemistry 51, 194–204 22122229 10.1021/bi201259y

[42] Beuve A., and Danchin A. (1992) From adenylate cyclase to guanylate cyclase: mutational analysis of a change in substrate specificity. J. Mol. Biol. 225, 933–938 10.1016/0022-2836(92)90093-Y 1351950

[43] Kleinboelting S., Diaz A., Moniot S., van den Heuvel J., Weyand M., Levin L. R., Buck J., and Steegborn C. (2014) Crystal structures of human soluble adenylyl cyclase reveal mechanisms of catalysis and of its activation through bicarbonate. Proc. Natl. Acad. Sci. U.S.A. 111, 3727–3732 10.1073/pnas.1322778111 24567411 PMC3956179

[44] Qi C., Sorrentino S., Medalia O., and Korkhov V. M. (2019) The structure of a membrane adenylyl cyclase bound to an activated stimulatory G protein. Science 364, 389–394 10.1126/science.aav0778 31023924

[45] Weichsel A., Kievenaar J. A., Curry R., Croft J. T., and Montfort W. R. (2019) Instability in a coiled-coil signaling helix is conserved for signal transduction in soluble guanylyl cyclase. Protein Sci. 28, 1830–1839 10.1002/pro.3707 31411784 PMC6739824

[46] Horst B. G., Yokom A. L., Rosenberg D. J., Morris K. L., Hammel M., Hurley J. H., and Marletta M. A. (2019) Allosteric activation of the nitric oxide receptor soluble guanylate cyclase mapped by cryo-electron microscopy. eLife 8, e50634 31566566 10.7554/eLife.50634 PMC6839917

[47] Kang Y., Liu R., Wu J.-X., and Chen L. (2019) Structural insights into the mechanism of human soluble guanylate cyclase. Nature 574, 206–210 10.1038/s41586-019-1584-6 31514202

[48] Ziegler M., Bassler J., Beltz S., Schultz A., Lupas A. N., and Schultz J. E. (2017) Characterization of a novel signal transducer element intrinsic to class IIIa/b adenylate cyclases and guanylate cyclases. FEBS J. 284, 1204–1217 10.1111/febs.14047 28222489

[49] Tucker C. L., Ramamurthy V., Pina A. L., Loyer M., Dharmaraj S., Li Y., Maumenee I. H., Hurley J. B., and Koenekoop R. K. (2004) Functional analyses of mutant recessive GUCY2D alleles identified in Leber congenital amaurosis patients: protein domain comparisons and dominant negative effects. Mol. Vis. 10, 297–303 15123990

[50] Friebe A., Wedel B., Harteneck C., Foerster J., Schultz G., and Koesling D. (1997) Functions of conserved cysteines of soluble guanylyl cyclase. Biochemistry 36, 1194–1198 10.1021/bi962047w 9063867

[51] Linder J. U. (2005) Substrate selection by class III adenylyl cyclases and guanylyl cyclases. IUBMB Life 57, 797–803 10.1080/15216540500415636 16393782

[52] Yan S. Z., Huang Z. H., Shaw R. S., and Tang W. J. (1997) The conserved asparagine and arginine are essential for catalysis of mammalian adenylyl cyclase. J. Biol. Chem. 272, 12342–12349 10.1074/jbc.272.19.12342 9139678

[53] DelProposto J., Majmudar C. Y., Smith J. L., and Brown W. C. (2009) Mocr: a novel fusion tag for enhancing solubility that is compatible with structural biology applications. Protein Expr. Purif. 63, 40–49 10.1016/j.pep.2008.08.011 18824232 PMC2936452

[54] Case D. A., Cerutti D. S., Cheatham T. E. III., Darden T. A., Duke R. E., Giese T. J., Gohlke H., Goetz A. W., Greene D., Homeyer N., Izadi S., Kovalenko A., Lee T. S., LeGrand S., Li P., et al (2016) Amber 2016, University of California, San Francisco, CA

[55] Hornak V., Abel R., Okur A., Strockbine B., Roitberg A., and Simmerling C. (2006) Comparison of multiple amber force fields and development of improved protein backbone parameters. Proteins Struct. Funct. Genet. 65, 712–725 10.1002/prot.21123 16981200 PMC4805110

[56] Maier J. A., Martinez C., Kasavajhala K., Wickstrom L., Hauser K. E., and Simmerling C. (2015) ff14SB: improving the accuracy of protein side chain and backbone parameters from ff99SB. J. Chem. Theory Comput. 11, 3696–3713 10.1021/acs.jctc.5b00255 26574453 PMC4821407

[57] Meagher K. L., Redman L. T., and Carlson H. A. (2003) Development of polyphosphate parameters for use with the AMBER force field. J. Comput. Chem. 24, 1016–1025 10.1002/jcc.10262 12759902

[58] Olsson M. H. M., Søndergaard C. R., Rostkowski M., and Jensen J. H. (2011) PROPKA3: consistent treatment of internal and surface residues in empirical p*K_a_* calculations. J. Chem. Theory Comput. 7, 525–537 10.1021/ct100578z 26596171

[59] Dolinsky T. J., Nielsen J. E., McCammon J. A., and Baker N. A. (2004) PDB2PQR: an automated pipeline for the setup of Poisson-Boltzmann electrostatics calculations. Nucleic Acids Res. 32, W665–W667 10.1093/nar/gkh381 15215472 PMC441519

[60] Jorgensen W. L., Chandrasekhar J., and Madura J. D. (1983) Comparison of simple potential functions for simulating liquid water. J. Chem. Phys. 79, 926–935 10.1063/1.445869

[61] Darden T., York D., and Pedersen L. (1993) Particle mesh Ewald: an N-log(N) method for Ewald sums in large systems. J. Chem. Phys. 98, 10089–10092 10.1063/1.464397

[62] Ryckaert J. P., Ciccotti G., and Berendsen H. J. C. (1977) Numerical integration of the cartesian equations of motion of a system with constraints: molecular dynamics of *n*-alkanes. J. Comput. Phys. 23, 327–341 10.1016/0021-9991(77)90098-5

[63] Roe D. R., and Cheatham T. E. 3rd (2013) PTRAJ and CPPTRAJ: software for processing and analysis of molecular dynamics trajectory data. J. Chem. Theory Comput. 9, 3084–3095 10.1021/ct400341p 26583988

[64] Wickham H. ggplot2: Elegant Graphics for Data Analysis. 2nd ed.; Springer-Verlag: New York, 2016; p 260 DOI:10.1007/978-3-319-24277-4

[65] Humphrey W., Dalke A., and Schulten K. (1996) VMD: visual molecular dynamics. J. Mol. Graph. 14, 33–38 10.1016/0263-7855(96)00018-5 8744570

[66] Doshi U., Holliday M. J., Eisenmesser E. Z., and Hamelberg D. (2016) Dynamical network of residue-residue contacts reveals coupled allosteric effects in recognition, catalysis, and mutation. Proc. Natl. Acad. Sci. U.S.A. 113, 4735–4740 10.1073/pnas.1523573113 27071107 PMC4855540

[67] Vu P. J., Yao X. Q., Momin M., and Hamelberg D. (2018) Unraveling allosteric mechanisms of enzymatic catalysis with an evolutionary analysis of residue-residue contact dynamical changes. ACS Catal. 8, 2375–2384 10.1021/acscatal.7b04263

[68] Grant B. J., Rodrigues A. P., ElSawy K. M., McCammon J. A., and Caves L. S. (2006) Bio3d: an R package for the comparative analysis of protein structures. Bioinformatics 22, 2695–2696 10.1093/bioinformatics/btl461 16940322

[69] Skjærven L., Yao X. Q., Scarabelli G., and Grant B. J. (2014) Integrating protein structural dynamics and evolutionary analysis with Bio3D. BMC Bioinformatics 15, 399 10.1186/s12859-014-0399-6 25491031 PMC4279791

[70] Csárdi G., and Nepusz T. (2006) The igraph software package for complex network research. InterJ. 1695 New England Complex Systems Institute: Cambridge, MA.

[71] Larkin M. A., Blackshields G., Brown N. P., Chenna R., McGettigan P. A., McWilliam H., Valentin F., Wallace I. M., Wilm A., Lopez R., Thompson J. D., Gibson T. J., and Higgins D. G. (2007) Clustal W and Clustal X version 2.0. Bioinformatics 23, 2947–2948 10.1093/bioinformatics/btm404 17846036

[72] Goujon M., McWilliam H., Li W., Valentin F., Squizzato S., Paern J., and Lopez R. (2010) A new bioinformatics analysis tools framework at EMBL–EBI. Nucleic Acids Res. 38, W695–W699 10.1093/nar/gkq313 20439314 PMC2896090

[73] Chojnacki S., Cowley A., Lee J., Foix A., and Lopez R. (2017) Programmatic access to bioinformatics tools from EMBL-EBI update: 2017. Nucleic Acids Res. 45, W550–W553 10.1093/nar/gkx273 28431173 PMC5570243

[74] Robert X., and Gouet P. (2014) Deciphering key features in protein structures with the new ENDscript server. Nucleic Acids Res. 42, W320–W324 10.1093/nar/gku316 24753421 PMC4086106

[75] McWilliam H., Li W., Uludag M., Squizzato S., Park Y. M., Buso N., Cowley A. P., and Lopez R. (2013) Analysis Tool Web Services from the EMBL-EBI. Nucleic Acids Res. 41, W597–W600 10.1093/nar/gkt376 23671338 PMC3692137

[76] Crooks G., Hon G., Chandonia J. M., and Brenner S. (2004) WebLogo: a sequence logo generator. Genome Res. 14, 1188–1190 10.1101/gr.849004 15173120 PMC419797

[77] Waterhouse A., Bertoni M., Bienert S., Studer G., Tauriello G., Gumienny R., Heer F. T., de Beer T. A. P., Rempfer C., Bordoli L., Lepore R., and Schwede T. (2018) SWISS-MODEL: homology modelling of protein structures and complexes. Nucleic Acids Res. 46, W296–W303 10.1093/nar/gky427 29788355 PMC6030848

[78] Guex N., Peitsch M. C., and Schwede T. (2009) Automated comparative protein structure modeling with SWISS-MODEL and Swiss-PdbViewer: a historical perspective. Electrophoresis 30, S162–S173 10.1002/elps.200900140 19517507

