# Supplementary material for: Synergistic mutations in soluble guanylyl cyclase (sGC) reveal a key role for interfacial regions in the sGC activation mechanism
Source: J Biol Chem. 2019 Oct 23;294(48):18451–64. doi: 10.1074/jbc.RA119.011010 (PMC6885636; doi:10.1074/jbc.RA119.011010)
Supplement: Supporting Information [file supp_294_48_18451__index.html]

Synergistic mutations in soluble guanylyl cyclase (sGC) reveal a key role for interfacial regions in the sGC activation mechanism — New mutations synergize to activate soluble guanylyl cyclase — Synergistic mutations in soluble guanylyl cyclase (sGC) reveal a key role for interfacial regions in the sGC activation mechanism — EDITORS' PICK: New mutations synergize to activate soluble guanylyl cyclase — Supporting Information 

# Synergistic mutations in soluble guanylyl cyclase (sGC) reveal a key role for interfacial regions in the sGC activation mechanism

## Supporting Information

- Supporting Information (to be published online) - Edited Supporting Methods, Figures, Tables
- Supporting Movies - Supporting MD movies
