# Supplementary material for: Synergistic mutations in soluble guanylyl cyclase (sGC) reveal a key role for interfacial regions in the sGC activation mechanism
Source: J Biol Chem. 2019 Oct 23;294(48):18451–64. doi: 10.1074/jbc.RA119.011010 (PMC6885636; doi:10.1074/jbc.RA119.011010)
Supplement: Supporting Information [file supp_RA119.011010_155745_2_supp_414169_pzkybn.pdf]

Supporting Information

Synergistic mutations in soluble guanylyl cyclase (sGC) reveal a key role for interfacial regions in the sGC activation mechanism

Childers K.C.[1,2], Yao X.Q.[3], Giannakoulias S.[1,4], Amason J.[1,5], Hamelberg D.[3], Garcin E.D.[1,*]

Supplementary Experimental Protocols.

Plasmids and gene construction

To replace the Spect^r gene present in the pCDF-Duet vector with the Amp^r gene from the pET-21a vector, we introduced flanking BmtI and BsaAI restriction sites to the Amp^r gene in the pET21a vector by site-directed mutagenesis. BmtI and BsaAI restriction enzymes and a Rapid DNA Ligation Kit (Sigma-Aldrich) were used to remove the Spect^r gene from pCDF-Duet and insert the Amp^r gene. Mutations were introduced using site-directed mutagenesis (Agilent). All sequences were confirmed by sequencing (GENEWIZ).

Supplementary Figures

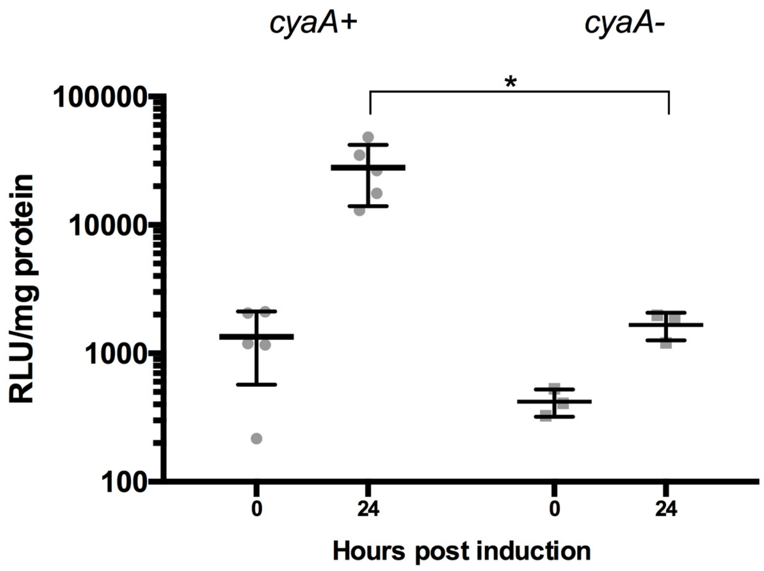

Figure S1. Luciferase activity from BL21(DE3) (*cyaA*+) and BL21(DE3) *cyaA*-deficient (*cyaA*-) cells transformed with pOPTXcGMPRE:LUC. All replicate data points (grey symbols) are represented as scatter dot plots. The mean and SD are represented as black lines. Statistical significance between *cyaA*$^{+}$ and *cyaA*$^{-}$ cells was analyzed using Student's t-test (*p < 0.02).

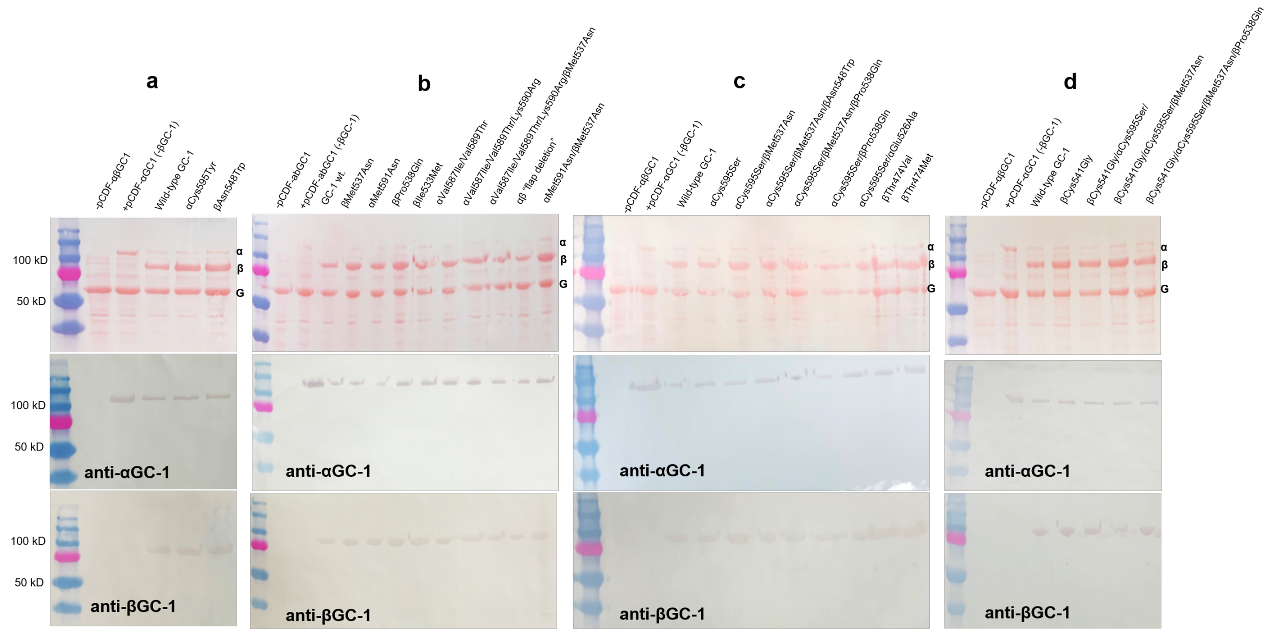

Figure S2. Western blots for expression of total protein using Ponceau Red stain (top), anti-αGC-1 (middle), and anti-βGC-1 (bottom) antibodies. Negative controls consisted of cells lacking either the αβGC-1 expression vector (-pCDF-αβGC1) or the βGC-1 gene (+pCDF-αGC1 (-βGC-1)). (a) Expression tests for wild-type, αCys595Tyr, and βAsn548Trp GC-1. (b) Expression test for wild-type GC-1 and dorsal flap variants. (c) Expression test for wild-type GC-1 and dimer interface variants. (d) Expression test for wild-type GC-1 and GTP-cleft variants. Protein bands corresponding to αGC-1 (α), βGC-1 (β), and GroEL (G) are indicated. Samples resuspended in SDS-PAGE loading buffer (60 mM Tris pH 6.8, 25% (v/v) glycerol, 2% (w/v) SDS, 5% (v/v) BME, and 0.2% (w/v) bromophenol blue) were incubated at 70 °C for 8 minutes and spun down at 10,000xg for 10 minutes at 4 °C. Proteins were transferred to a nitrocellulose membrane (BioRad) and detected using Ponceau Red. Membranes were blocked using a 4% (w/v) milk solution and exposed to either rabbit anti-αGC-1 (residues 673-690, Sigma-Aldrich) or rabbit anti-βGC-1 (residues 605-619, Sigma-Aldrich) primary antibodies and goat anti-rabbit (BioRad) secondary antibodies coupled to alkaline phosphatase. Bands were revealed using NBT and BCIP reagents (Thermo Fisher).

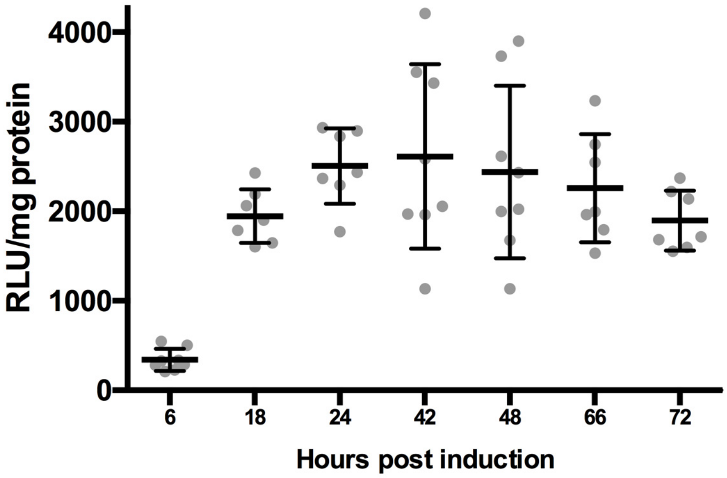

Figure S3. Background luciferase activity measured in BL21(DE3) *cyaA*- cells transformed with pOPTXcGMPRE:LUC, pGRO7, and pCDF-αGC-1 (lacking βGC-1). All replicate data points for each post-induction time point (grey symbols) are represented as scatter dot plots. The mean and SD are represented as black lines.

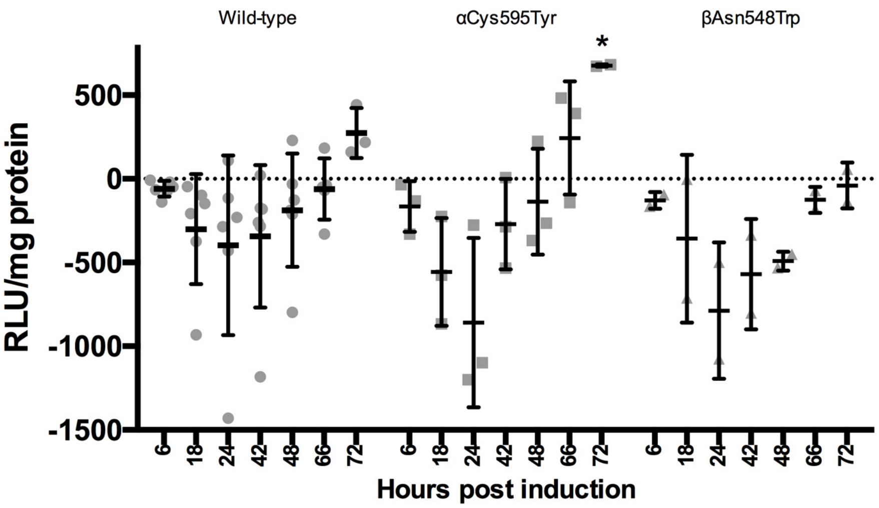

Figure S4. Luciferase activity measured in BL21(DE3) *cyaA-* cells transformed with pOPTXcGMPRE:LUC, pGRO7, and pCDF-αβGC-1 (wild-type, αCys595Tyr, or βAsn548Trp GC-1). All replicate data points for each post-induction time point (grey symbols) are represented as scatter dot plots. The mean and SD are represented as black lines.. Statistical significance between wild-type and mutant GC-1 cells at 72-hour post induction was calculated using the Student's *t*-test (*$p < 0.05$).

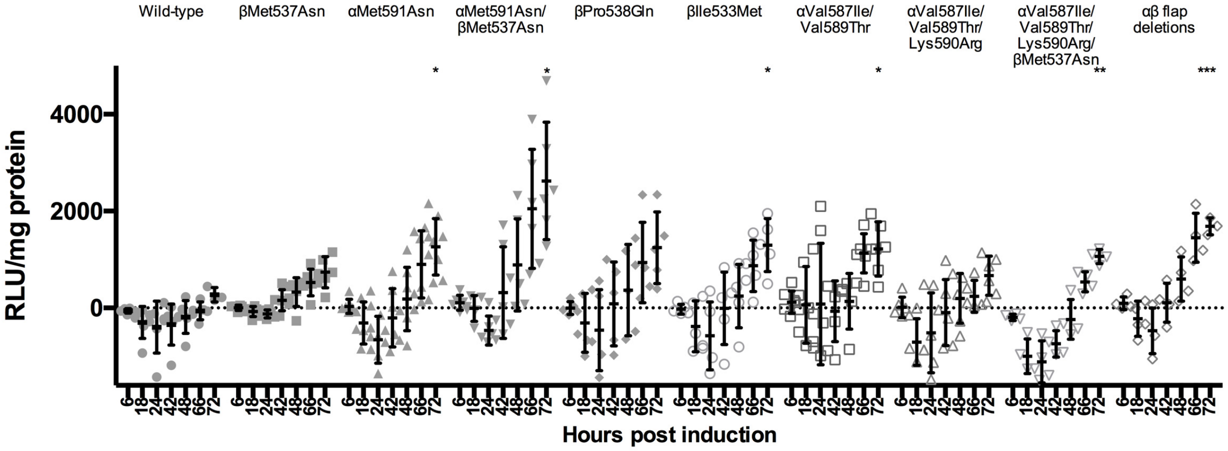

Figure S5. Luciferase activity measured in BL21(DE3) *cyaA-* cells transformed with pOPTXcGMPRE:LUC, pGRO7, and pCDF-αβGC-1 (wild-type or dorsal flap variants). All replicate data points for each post-induction time point (grey symbols) are represented as scatter dot plots. The mean and SD are represented as black lines. Statistical significance between wild-type and mutant GC-1 cells at 72-hour post induction was calculated using the Student's *t*-test (*$p < 0.05$; **$p < 0.001$; ***$p < 0.0005$).

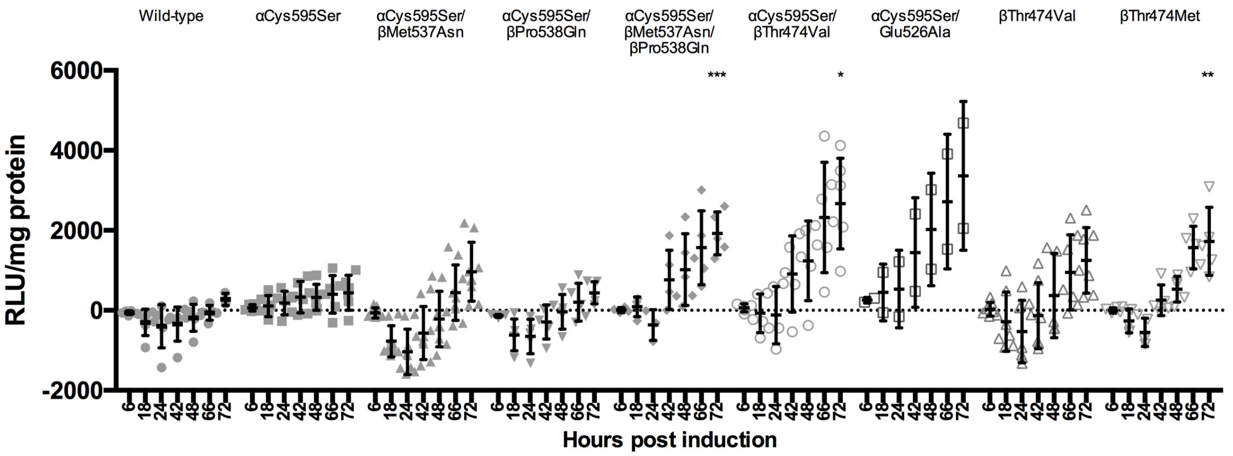

Figure S6. Luciferase activity measured in BL21(DE3) *cyaA-* cells transformed with pOPTXcGMPRE:LUC, pGRO7, and pCDF-αβGC-1 (wild-type or interfacial variants). All replicate data points for each post-induction time point (grey symbols) are represented as scatter dot plots. The mean and SD are represented as black lines. Statistical significance between wild-type and mutant GC-1 cells at 72-hour post induction was calculated using the Student's *t*-test (**p* < 0.01; ***p* < 0.05; ****p*<0.005).

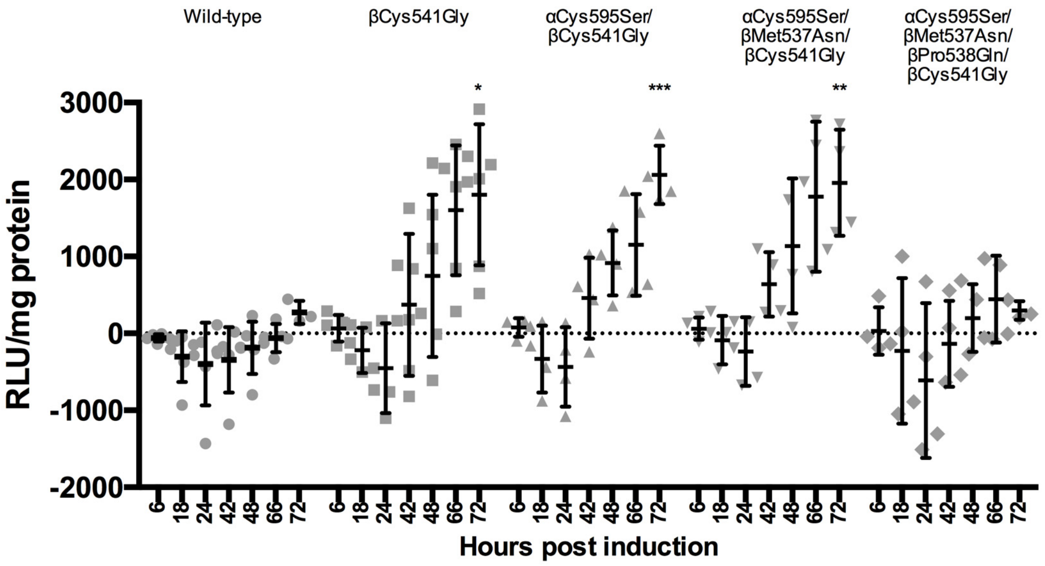

Figure S7. Luciferase activity measured in BL21(DE3) *cyaA-* cells transformed with pOPTXcGMPRE:LUC, pGRO7, and pCDF-αβGC-1 (wild-type or GTP-cleft variants). All replicate data points for each post-induction time point (grey symbols) are represented as scatter dot plots. The mean and SD are represented as black lines. Statistical significance between wild-type and mutant GC-1 cells at 72-hour post induction was calculated using the Student's *t*-test (*p<0.05; ***p* < 0.01; ****p* < 0.001).

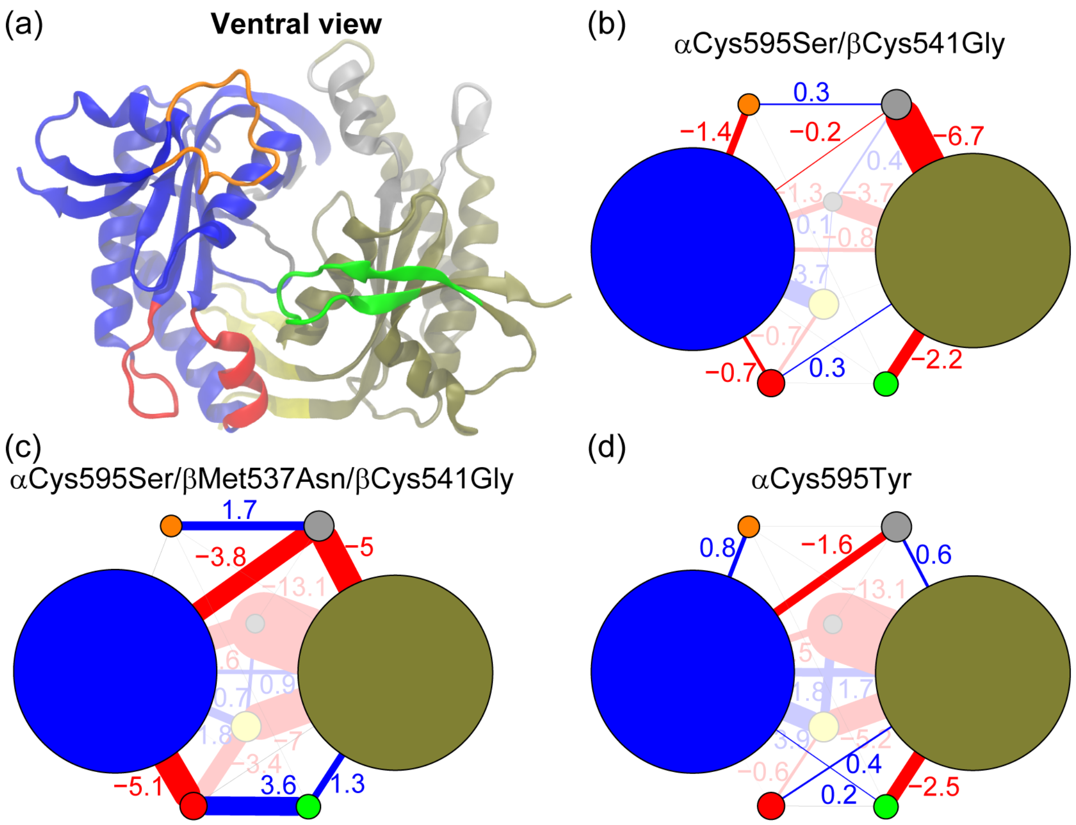

Figure S8. Function-related rearrangements of contacts/interactions between residue communities or segments on the ventral side. (a) Eight communities (colored regions) were identified using the difference contact network analysis method. (b-d) Community-community difference contact networks (with respect to wild-type αβGC^{cat}) for the three mutants. Communities are represented by colored vertices (as in panel a), with radius of vertex proportional to the number of residues in the community. The line linking vertices describes the sum of contact probability changes (denoted by *df*) between the communities. Blue and red lines indicate positive and negative changes (with *df* labeled), respectively, and the line width is determined by |*df*|. Vertices and lines corresponding to contact changes on the dorsal side are blurred for clarity.

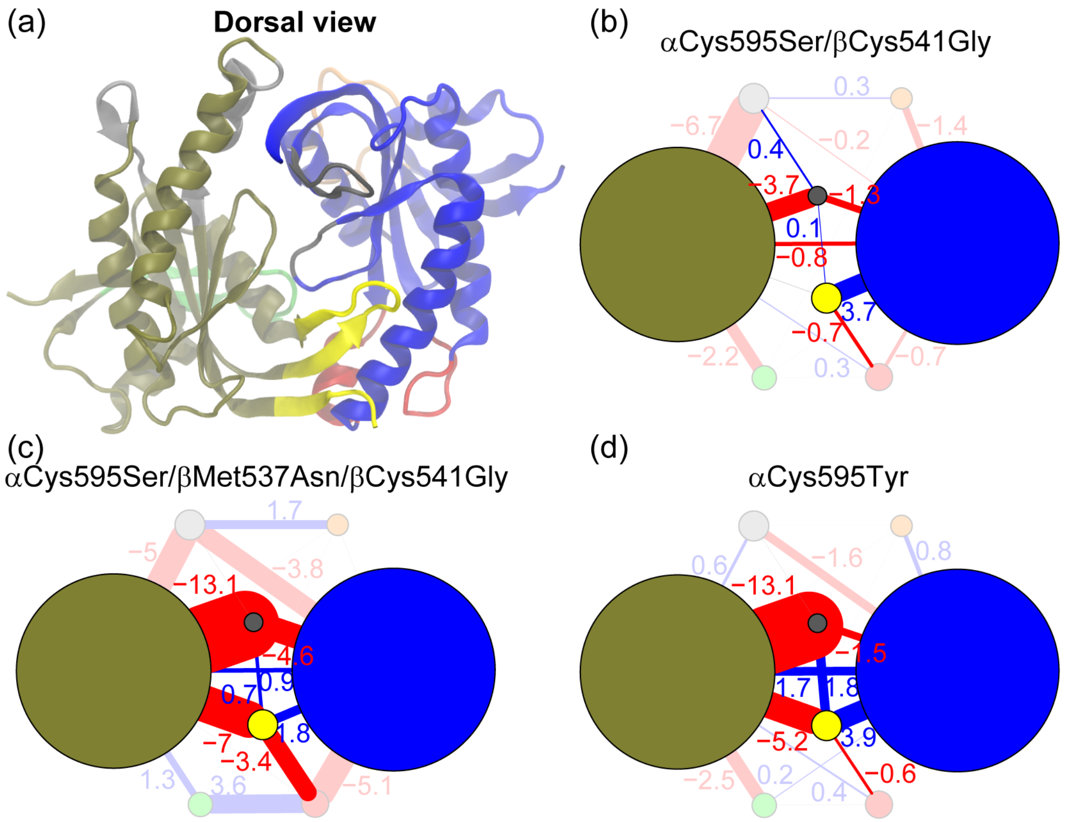

Figure S9. Function-related rearrangements of contacts/interactions between residue communities or segments on the dorsal side. (a) Eight communities (colored regions) were identified using the difference contact network analysis method. (b-d) Community-community difference contact networks (with respect to wild-type αβGC^{cat}) for the three mutants. Communities are represented by colored vertices (as in panel a), with radius of vertex proportional to the number of residues in the community. The line linking vertices describes the sum of contact probability changes (denoted by *df*) between the communities. Blue and red lines indicate positive and negative changes (with *df* labeled), respectively, and the line width is determined by |*df*|. Vertices and lines corresponding to contact changes on the ventral side are blurred for clarity.

Supplementary Movie S1

The collective motions represented by PC1 contained an obvious relative rotation of the two subunits.

Supplementary Movie S2.

The collective motions represented by PC2 mainly contained local conformational rearrangements in both ventral and dorsal surfaces.

Supplementary Table S1. List of plasmids for reporter assay. [a]Oligopeptide transporter X cyclic guanosine 3',5'-monophosphate reporter gene: luciferase.

Plasmid Name	Gene	Promoter	Antibiotic Resistance	Origin of Replication
pOPTXcGMPRE:LUC[a]	Luciferase	*OPTXcGMPRE*	Kan^r	pBR322
pGro7	GroEL/ES	*araB*	$Chlor^r$	p15A
pCDF-αβGC1	αGC-1/βGC-1	*T7*	Amp^r	CloDF13
pCDF-αGC1	αGC-1	*T7*	Amp^r	CloDF13

Supplementary Table S2

Extracellular cAMP levels from BL21(DE3) *cyaA*+ and BL21(DE3) *cyaA*- (*cyaA* deficient) cells. Samples were taken after 24 hours of cell growth and were plated in duplicate. Error represents the standard error of the mean from three or more independent experiments. [a]Not detectable.

Cells	pmol cAMP/mg protein ($\times 10^5$)
BL21(DE3) *cyaA*+	2.58 ± 0.89
BL21(DE3) *cyaA*-	N/D[a]

Supplementary Table S3

Intracellular and extracellular cGMP levels from BL21(DE3) *cyaA-* cells transformed with pOPTXcGMPRE:LUC, pGRO7, pCDF-αGC-1 (lacking βGC-1), or pCDF-αβGC-1. The cGMP levels were measured after 72 hours of cell growth and were plated in duplicate. Error represents the standard error of the mean from three or more independent experiments. * indicates one experimental replicate

Sample	Intracellular pmol cGMP/mg protein	Extracellular pmol cGMP/mg protein
αGC-1 (-βGC-1)*	4.8	894
Wild-type αβGC-1	6.2 ± 1.4	1874 ± 458
αCys595Ser GC-1	5.8 ± 1.4	12147 ± 1961
βMet537Asn GC-1	4.8 ± 1.6	4145 ± 895
αCys595Ser/βMet537Asn/βCys541Gly GC-1	5.0 ± 1.0	28604 ± 4627
